# DFT investigation of efficient hydrogen storage utilizing Li and Na decorated co-doped graphene (B/N)

**DOI:** 10.1038/s41598-025-14088-8

**Published:** 2025-08-19

**Authors:** N. N. Mostafa, Kamal A. Soliman, S. M. Abd El Haleem, W. S. Abdel Halim

**Affiliations:** 1 Department of Chemistry, Faculty of Science, Zagazig University, P.O.Box 44519, Zagazig, Egypt; 2 Department of Chemistry, Faculty of Science, Benha University, P.O. Box 13518, Benha, Egypt

**Keywords:** Hydrogen storage, Co-doped graphene, Li and na decoration, DOS, PDOS, Gravimetric density, Desorption temperature, Theoretical chemistry, Energy

## Abstract

This study investigates the hydrogen storage capacity of co-doped graphene with non-bonded B and N atoms (BC_4_N) using density functional theory (DFT). The optimized structure reveals the introduction of co-doping ripples the surface, enhancing potential hydrogen storage applications. The adsorption behavior of Li and Na atoms on the BC_4_N surface is examined, demonstrating a higher binding energy, surpassing their cohesive energies. Density of State (DOS), Partial Density of State (PDOS), and charge transfer analyses indicate electron donation from Li and Na to BC_4_N monolayer, establishing BC_4_N as an electron acceptor. The investigation extends to H_2_ adsorption on Li/BC_4_N and Na/BC_4_N systems, revealing a non-dissociative form and a cooperative effect with increasing H_2_ molecules. The hydrogen storage gravimetric density is calculated, and desorption temperatures are determined, highlighting the potential of Li/BC_4_N and Na/BC_4_N as promising candidates for efficient hydrogen storage.

**Supplementary Information:**

The online version contains supplementary material available at 10.1038/s41598-025-14088-8.

## Introduction

The focus on hydrogen as an ideal energy resource has grown due to its favorable characteristics, including high energy density, renewability, and friendly to the environment. Its potential to replace fossil fuels has led to a significant emphasis on developing a stable and cost-effective storage system^1–6^. The US Department of Energy has a target for hydrogen storage capacity of 5.5 wt% of weight density^7^, emphasizing the importance of achieving optimal binding energy for effective hydrogen recycling at near-ambient conditions^8,9^. Various carbon nanostructures, such as carbon nanotubes, and graphene have been investigated for their hydrogen adsorption capabilities, driven by their reversibility, fast kinetics, and high capacities^10–15^. The interest in the processes of adsorption and desorption with carbon-based materials from the intrinsic advantageous properties these materials possess. These include low density, high chemical stability, and the capacity to manipulate pore structures and surface areas, rendering them compelling options for hydrogen storage technologies^16^. Among carbon-based materials, the focus of research has notably turned towards graphene-based carbon materials. This shift is primarily driven by the extraordinary features exhibited by graphene, such as an exceptionally high specific surface area, mechanical flexibility, robustness, and a lightweight composition. These attributes position graphene as a promising candidate for hydrogen storage devices, emphasizing its potential for practical applications^17^.

Furthermore, graphene an exceptional two-dimensional carbon material known for its distinctive properties has become a focal point of research interest due to recent advancements in its large-scale production^18^. Apart from its prevalent applications in electronics, graphene plays a pivotal role in sensor production and serves as a molecular adsorbent. While previous studies extensively explored graphene’s efficacy in adsorbing various small molecules, its potential as hydrogen (H_2_) adsorbent is limited, and hydrogen passivation may even compromise the mechanical properties of graphene^19^. To address these challenges, sophisticated techniques are necessary for effective hydrogen uptake. Some studies have opted for innovative approaches, such as utilizing curved graphene or graphene hollows to enhance interactions with H_2_/adsorbent^20^. On the other hand, alternative strategies involve employing planar graphene sheets with doped heteroatoms. Transition metals such as titanium (Ti)^21^, iron (Fe)^22^, nickle (Ni)^23^, palladium (Pd)^24^, and platinum (Pt)^25^ can be useful for this purpose.

Ao et al. explored Al-doped porous graphene by DFT study. Their investigations demonstrated the efficient adsorption of H_2_ molecules by such structures with adsorption energy of − 0.41 eV^15^. Huo et al. employed theoretical methods to investigate Ti-decorated boron-doped porous graphene in adsorbing H_2_ molecules^26^. They investigated that boron-doped porous graphene decorated with titanium atoms can consistently capture sixteen hydrogen molecules, achieving a gravimetric hydrogen uptake of 8.58 wt%.

Researchers conducted both experimental and theoretical investigations on graphene to enhance hydrogen storage capacity. The studies revealed that modifying the electronic properties of dispersed carbon-based materials through doping with nonmetallic elements (such as B and N) or intentionally creating vacancies could be a promising strategy. This approach has been shown to enhance the adsorption performance of hydrogen according to various works^27,28^. Yang et al.^29^ demonstrated that, under conditions of 298 K and 10 Mpa, the hydrogen storage capacity increased to 1.2 wt% through hydrogen spillover on ruthenium-decorated boron-doped microporous carbon. In the study conducted by Wu and colleagues^30^, they observed that B-doped graphene has a superior ability to adsorb hydrogen compared to pristine graphene. This increased adsorption capability is attributed to the presence of low activation barriers, particularly under ambient conditions. Furthermore, Lee et al.^31^ conducted research on the utilization of Li-decorated graphene for hydrogen storage. In this study, they introduced nitrogen defects that are experimentally feasible to enhance the overall performance of the hydrogen storage system.

This study introduces an approach by suggesting a unique arrangement of graphene sheets that co-doped with boron and nitrogen and featuring Li, and Na atoms to enhance hydrogen storage. The B, and N atoms co-doped graphene are non-bonded that be more efficient catalyst when compared to bonded B-N co-doped graphene^32,33^.To assess the influence of this co-doped structure on hydrogen adsorption, the study utilizes first-principle analyses based on density functional theory (DFT).

## Results and discussion

### Structural properties of co-doped graphene (B and N atom), decorated by li, and Na atom

**Fig. 1:**
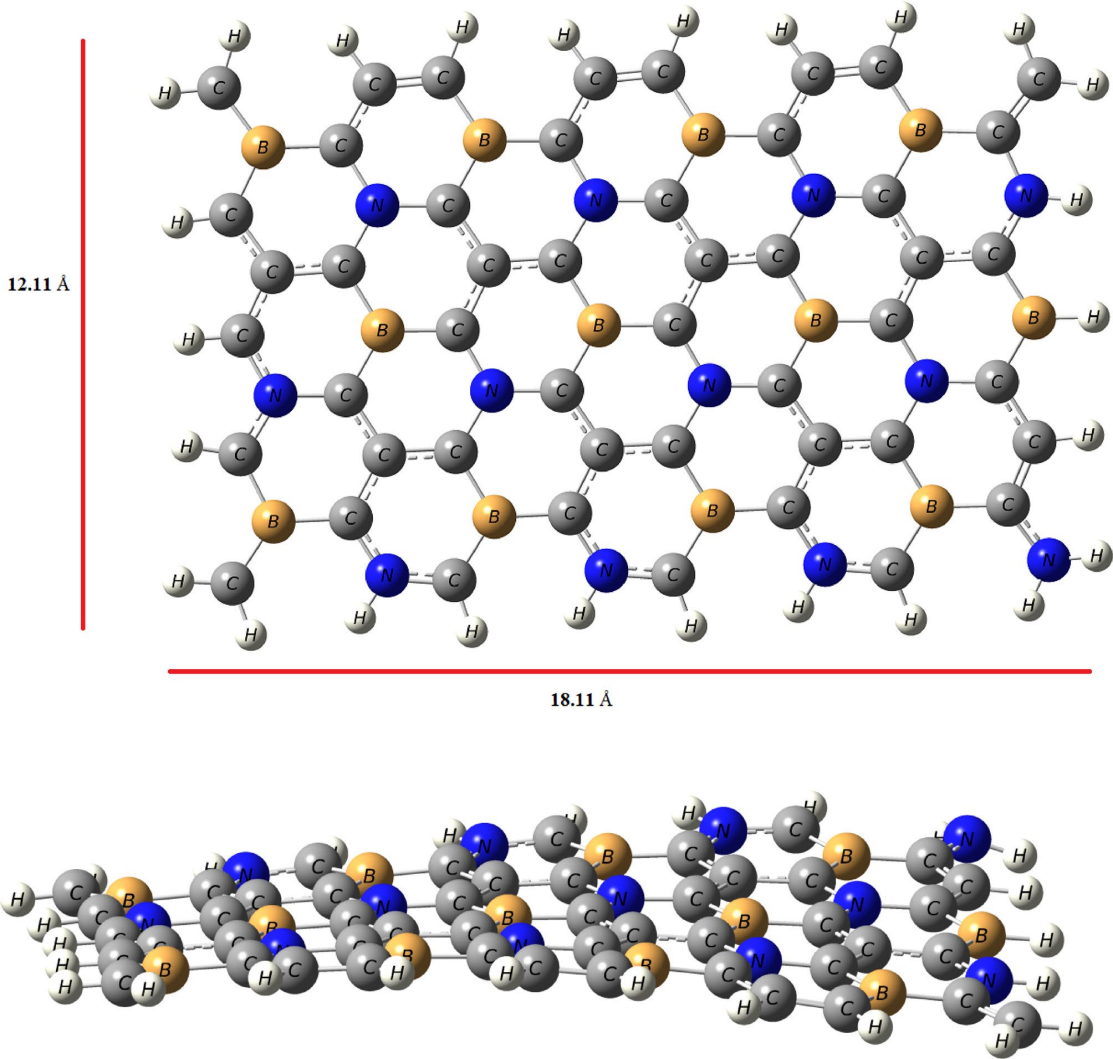
The optimized structure of B, and N co-doped graphene; top- (up) and side- (down) view.

**Fig. 2:**
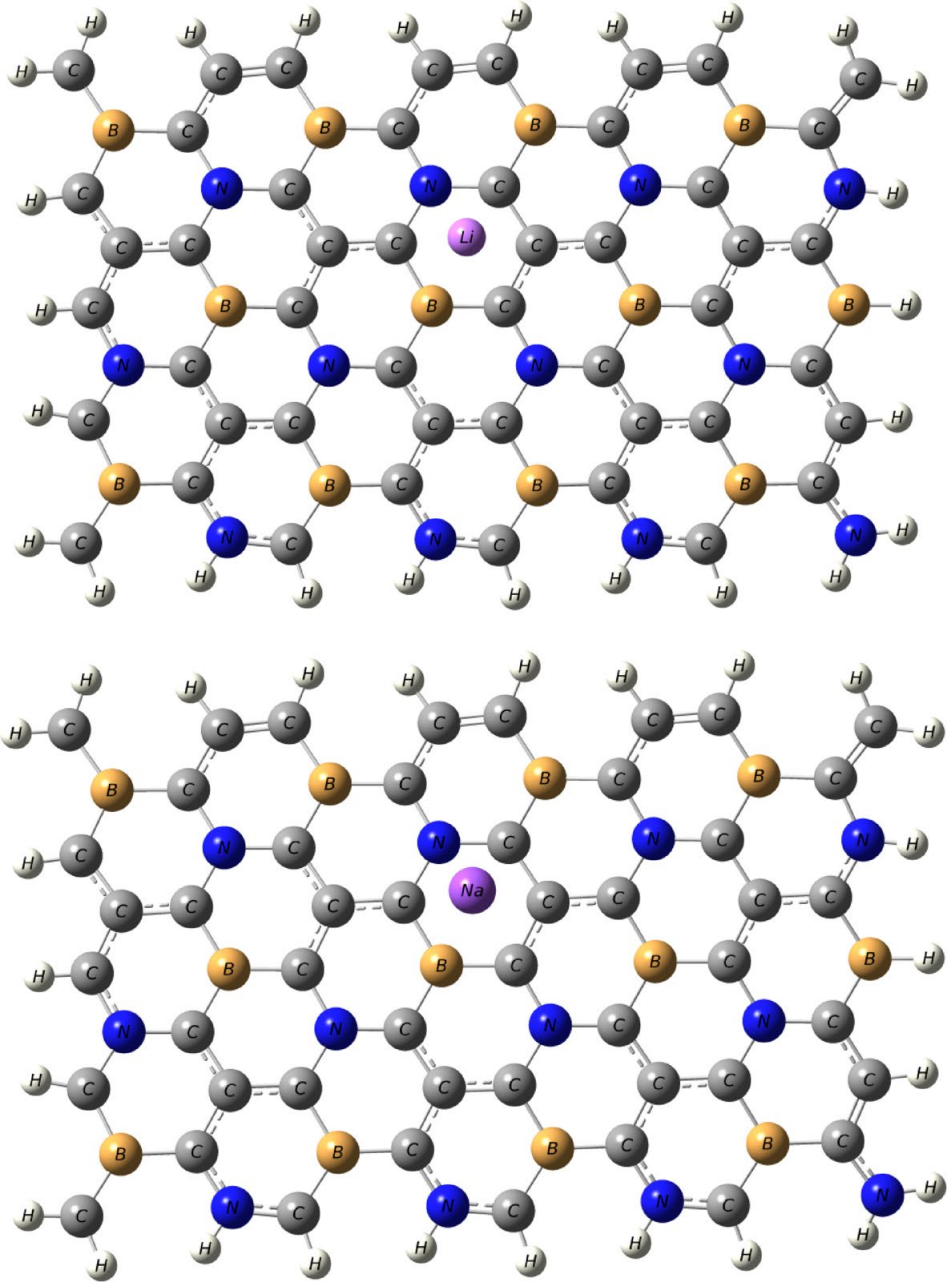
The optimized structure of Li “up”, and Na “lower” decorated BC_4_N monolayer.

To explore the hydrogen storage capacity of co-doped graphene with B, and N atom that is non-bonded, the optimized geometry of this system (BC_4_N) is shown in Fig. 1, and the corresponding data are compiled in Table 1. A vibrational frequency calculation was also performed on the optimized BC4N sheet (B3LYP/6-31G(d, p)); no imaginary frequencies were found, confirming that the structure shown in Fig. 1 corresponds to a true local minimum. As seen in Table 1, the bond lengths between B-C, C-C, and C-N are 1.50 Å, 1.43 Å, and 1.41 Å respectively. The structure of pristine graphene is flat and by co-doped with B, and N atom that are non-bonded, the presence of ripples appears that is may be effective in hydrogen storage application. Initially, the study of binding of single atom decorated the surface such as Li, and Na atoms was determined. As seen in Fig. 2, the optimized Li, and Na atom on the BC_4_N surface are prefer to be adsorbed on hollow center of the hexagon and Cartesian coordinates for the Li/BC_4_N and Na/BC_4_N are shown in Table S1 and S2. This corresponds well with the preferred site for Li atom adsorption on both pure graphene and porous graphene^2,34,35^. In the optimized structures, the distance between Li/Na and nearest carbon atom is 2.17/2.53 Å, respectively as presented in Table 1. The binding energies for Li and Na atom over the surface were found to be −2.51 eV, and − 1.95 eV respectively that is higher than their cohesive energies^36,37^. The results show that co-doped graphene with B, and N atom has the capacity to significantly enhance the binding energy of Li, and Na atoms, exceeding the cohesive energy of Li and Na. This intentional effect results in the suppression of Li, and Na adatom clustering on BC_4_N system. Therefore, co-doped graphene with B, and N atom demonstrates significant potential for hydrogen storage due to its characteristic of maintaining geometric stability.

**Table 1:** Equilibrium distance (d, Å), binding energies of li, and Na decorated BC4N monolayer and the charge.

System	d (Å)	E_b_(eV)	Q_NBO_(e)
BC_4_N	1.50 (B-C) 1.43 (C-C) 1.41 (N-C)		
Li/BC_4_N	2.17 (Li-C)	−2.51	0.90 (Li)
Na/BC_4_N	2.53 (Na-C)	−1.95	0.95 (Na)

**Fig. 3:**
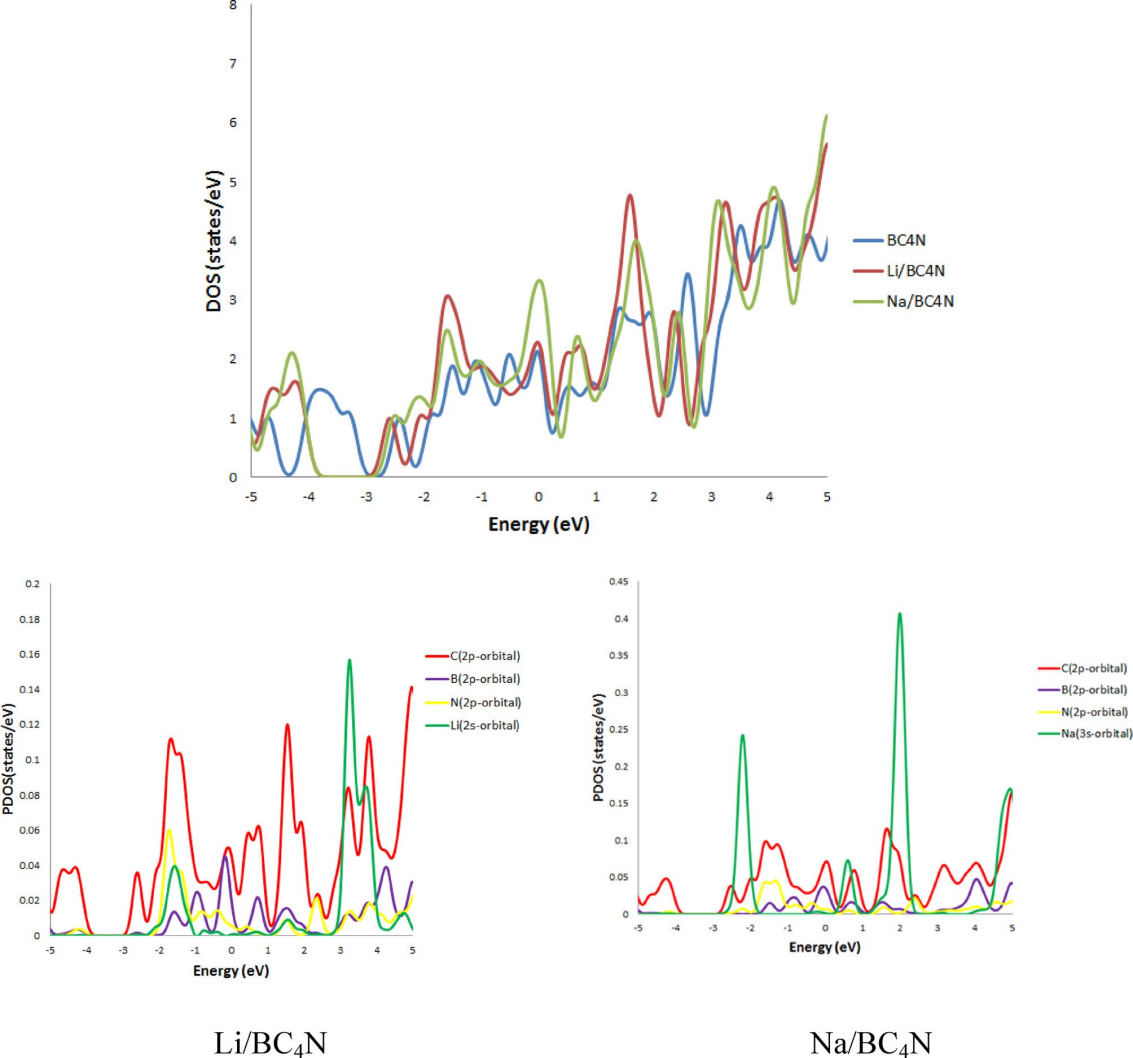
Total DOS and PDOS of BC_4_N and Li, Na atom decorated BC_4_N surfaces.

 The interaction of the adsorbed Li, and Na atom on the BC_4_N system were also studied by calculating the Density of State (DOS), Partial Density of State (PDOS), and charge transfer that obtained from natural bond orbital (NBO). The analysis reveals an electronic charge transfer from the Li, and Na adatom to the substrate, as illustrated in Table 1. Essentially, the adsorbent functions as an electron acceptor in relation to the Li, and Na adatom. Based on these results, it can be inferred that BC_4_N systems exhibit a higher binding energy for Li, and Na adatoms on the surface. Despite the fact that the charge transfer from Na to BC_4_N surface is greater than that from Li, the binding energy of Li on BC4N is greater. This seemingly strange finding can be explained by the fact that both Li and Na are electrostatically attracted to BC_4_N π-electrons. Li atom experiences stronger electrostatic attraction than Na atom due to its smaller size and higher nuclear charge, and the smaller size of Li allows its orbitals to overlap more efficiently with BC4N π-orbitals, developing stronger covalent bonding, whereas Na atom’s larger size makes its orbital overlap with BC4N less optimal, limiting the covalent contribution to its binding. By analyzing the density of states (DOS) and projected density of states (PDOS) as seen in Fig. 3, The observation that peaks in the density of states (DOS) increase after decorating BC4N with Li, and Na atom. When Li and Na atom are deposited on BC4N surface, they interact with its π-electron system and this interaction can be understand by PDOS. The Fig. 3, when Li atom sit on hollow site, its 2s-orbital of Li atom directly overlap with the 2p orbitals of the surrounding C, B, and N atom in the BC4N surface at energies − 1.64 eV, 1.39 eV, and 3.25 eV. Also, Na decorated BC4N surface show overlap between 3s-orbital of Na atom with 2p orbitals of the C, B, nad N atom in the BC4N surface at energies 0.55 eV, at −2.24 eV and 1.99 eV there is overlap between 3s-orbital of Na atom with 2p-orbitals of N and C atom of the BC4N surface.

### Hydrogen storage properties

**Fig. 4:**
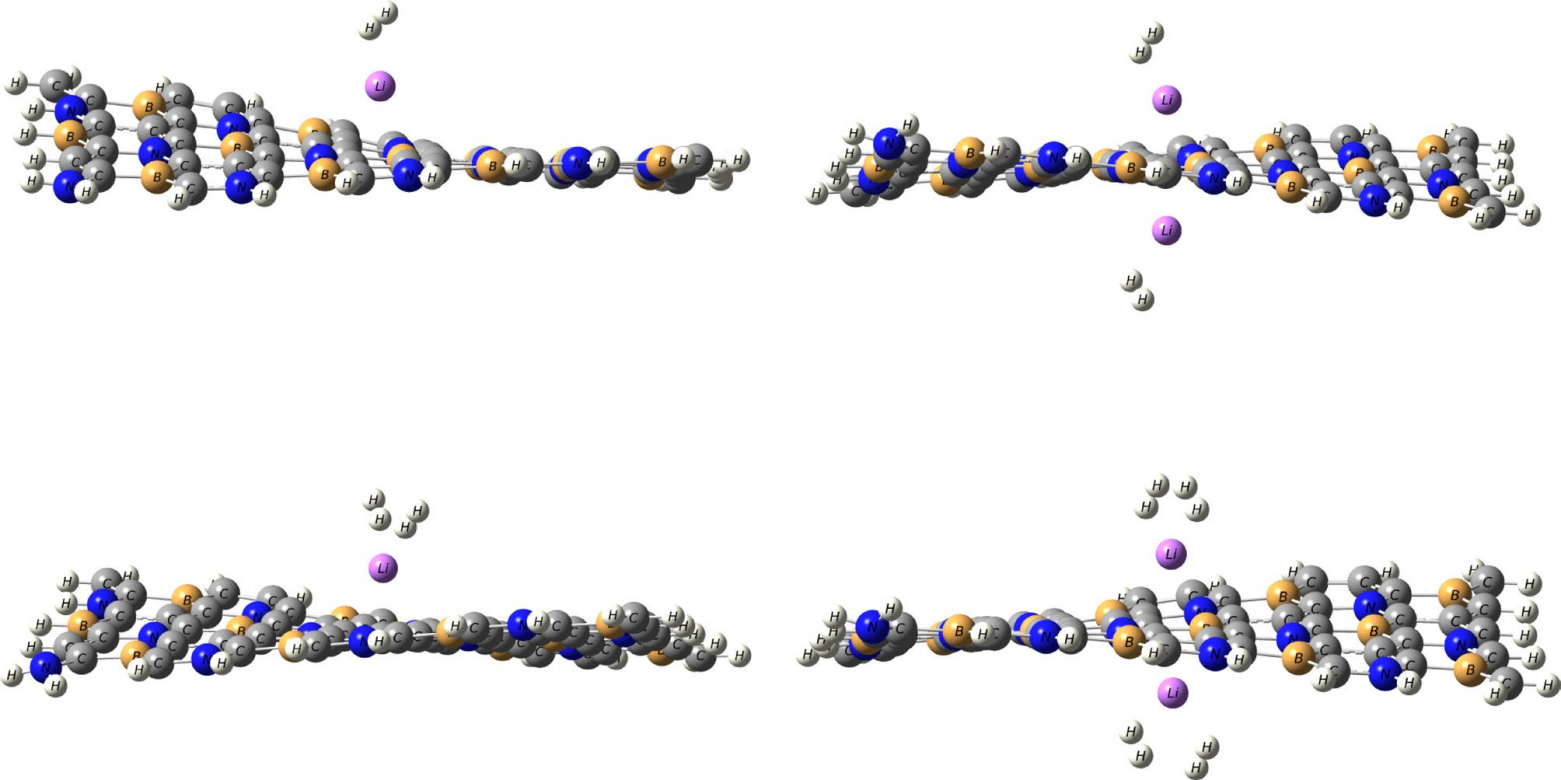

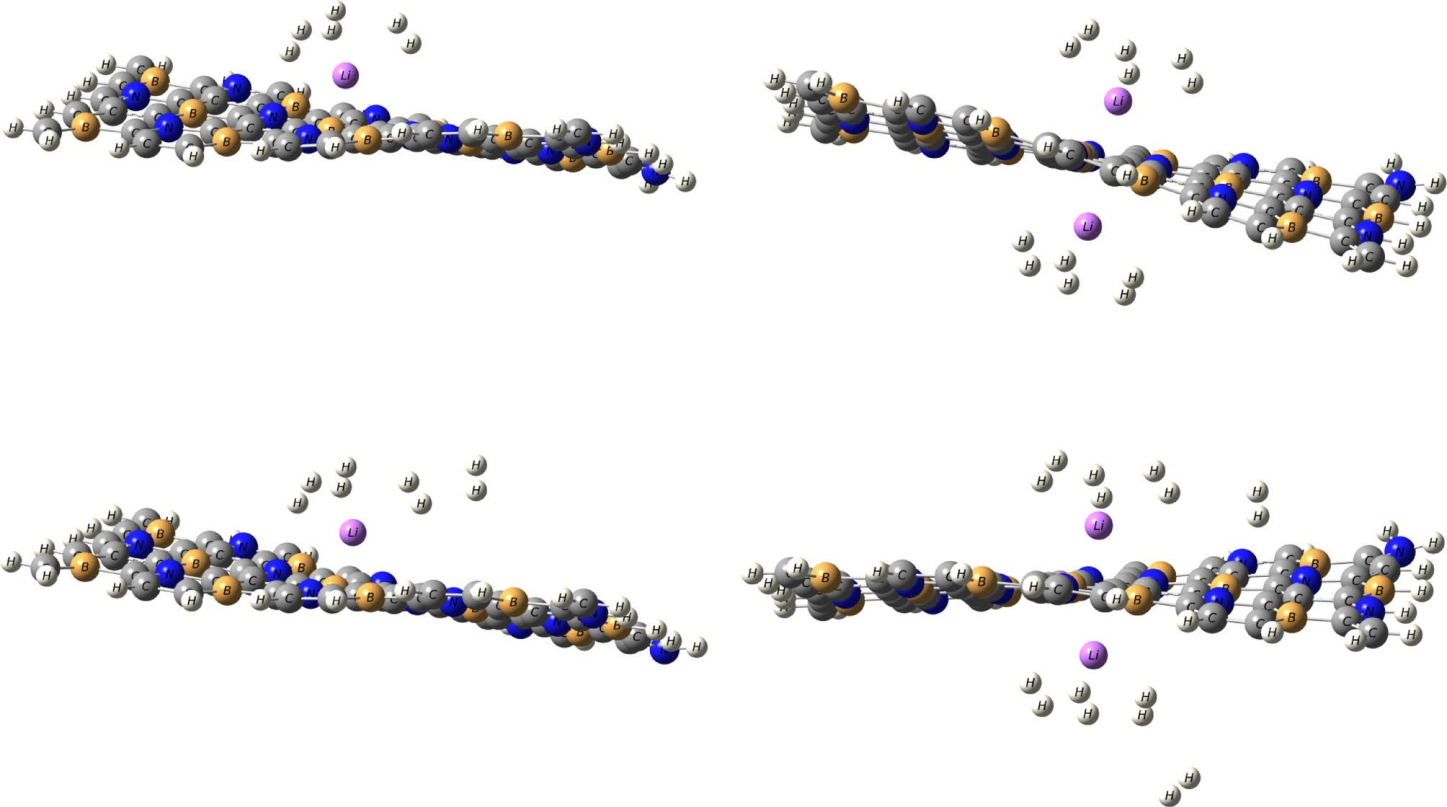
Optimized structures of H_2_ molecules on Li/BC_4_N monolayer.

**Fig. 5:**
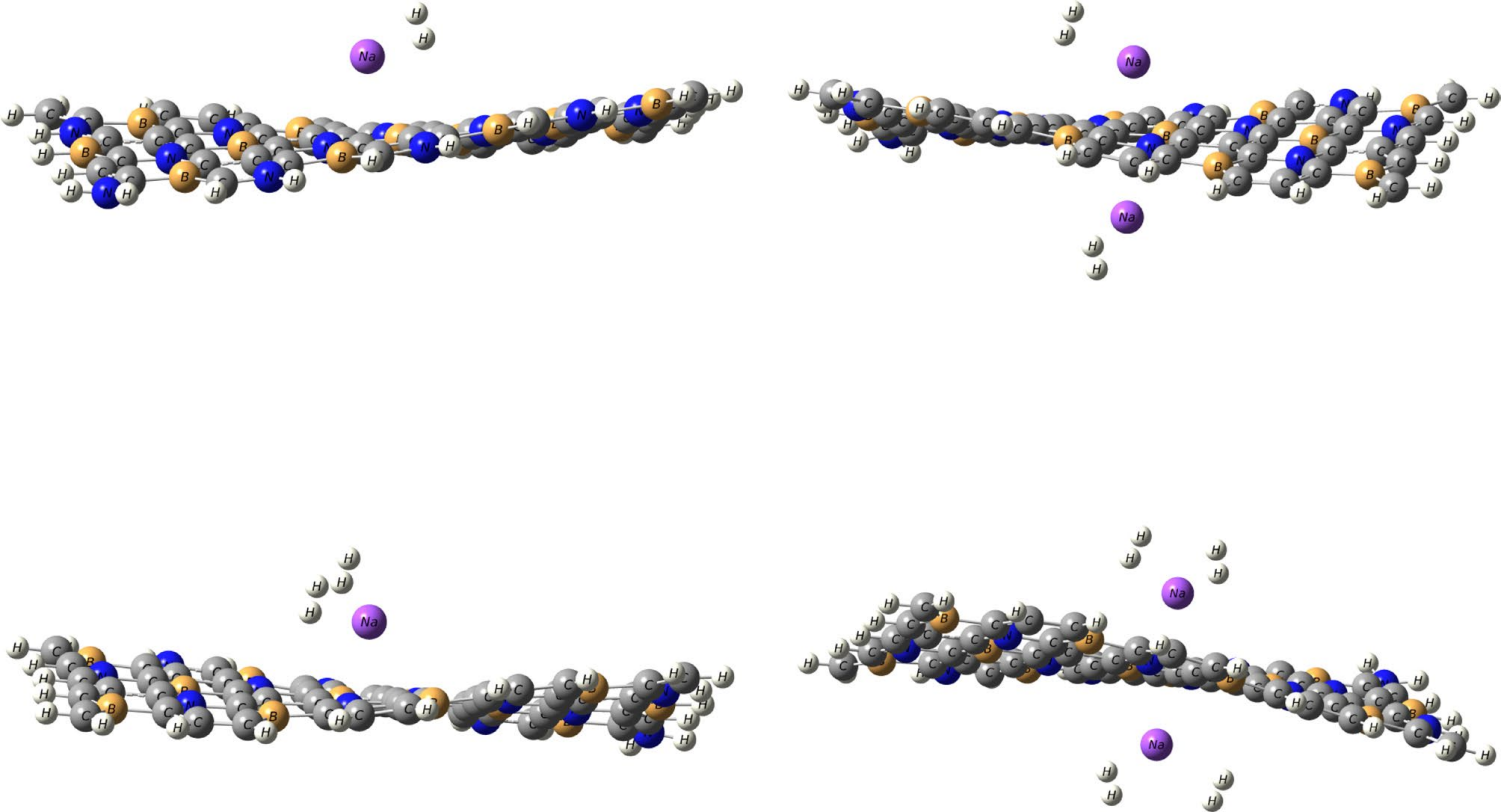

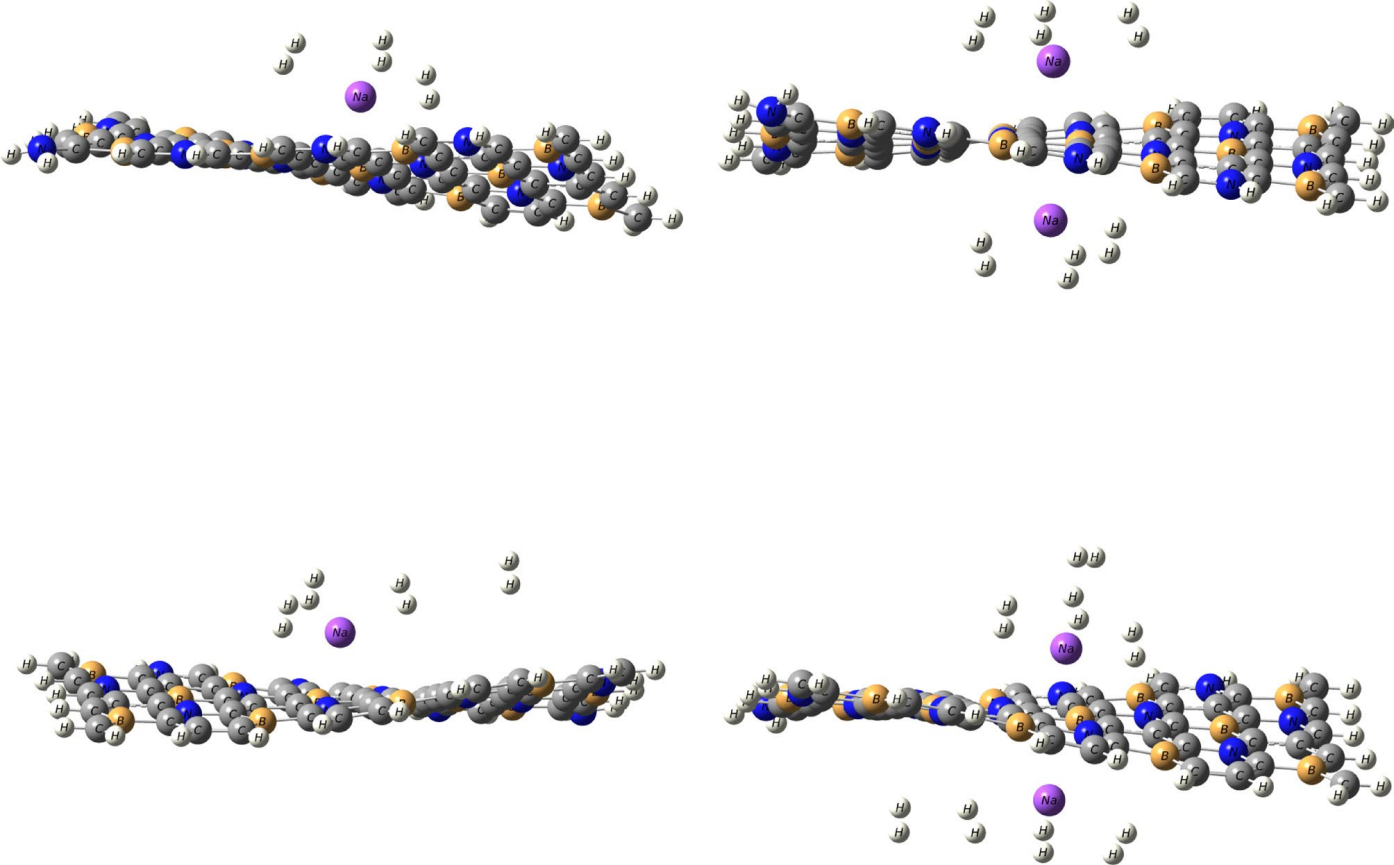
Optimized structures of H_2_ molecules on Na/BC_4_N monolayer.

**Fig. 6:**
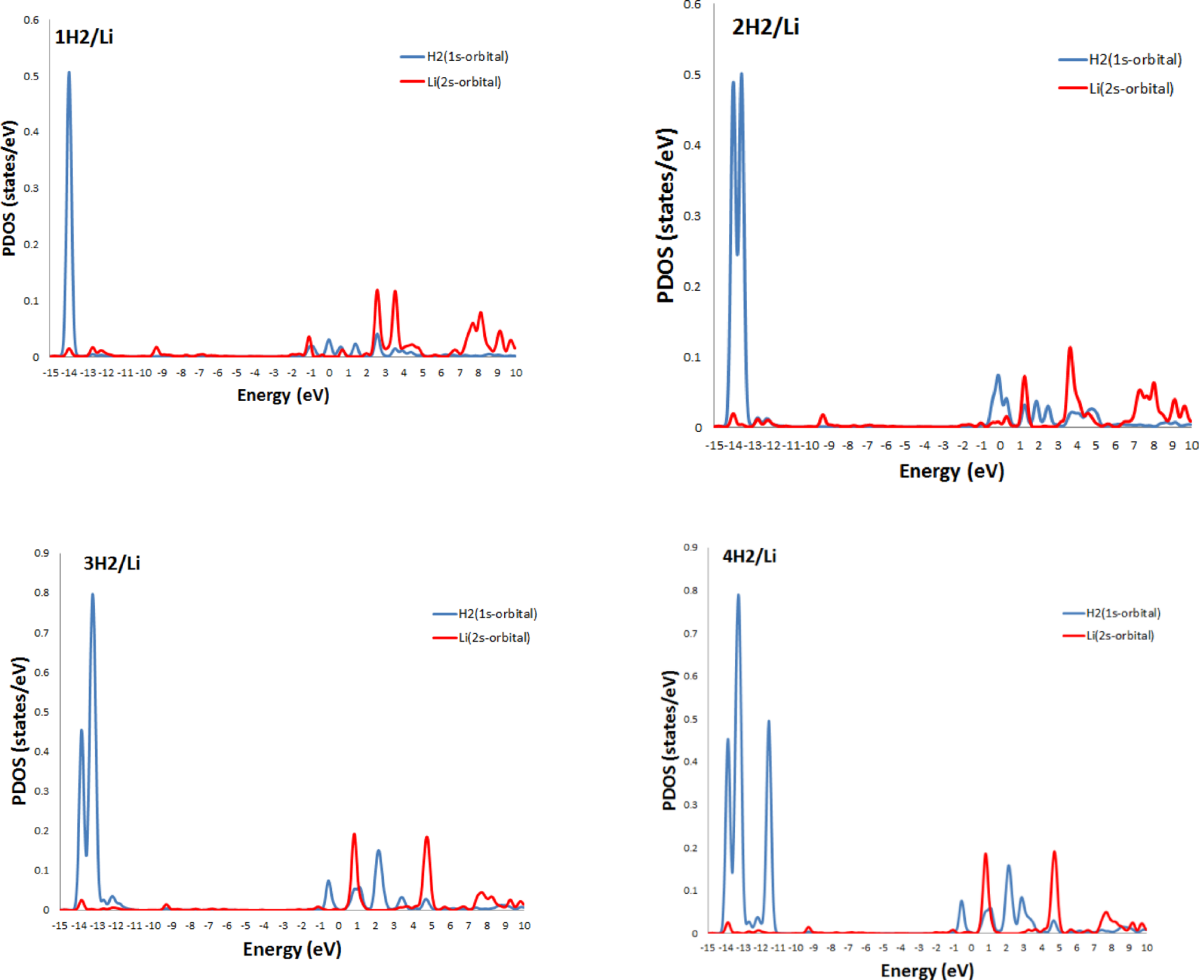
PDOS of H_2_ molecules on Li/BC_4_N monolayer.

**Fig. 7:**
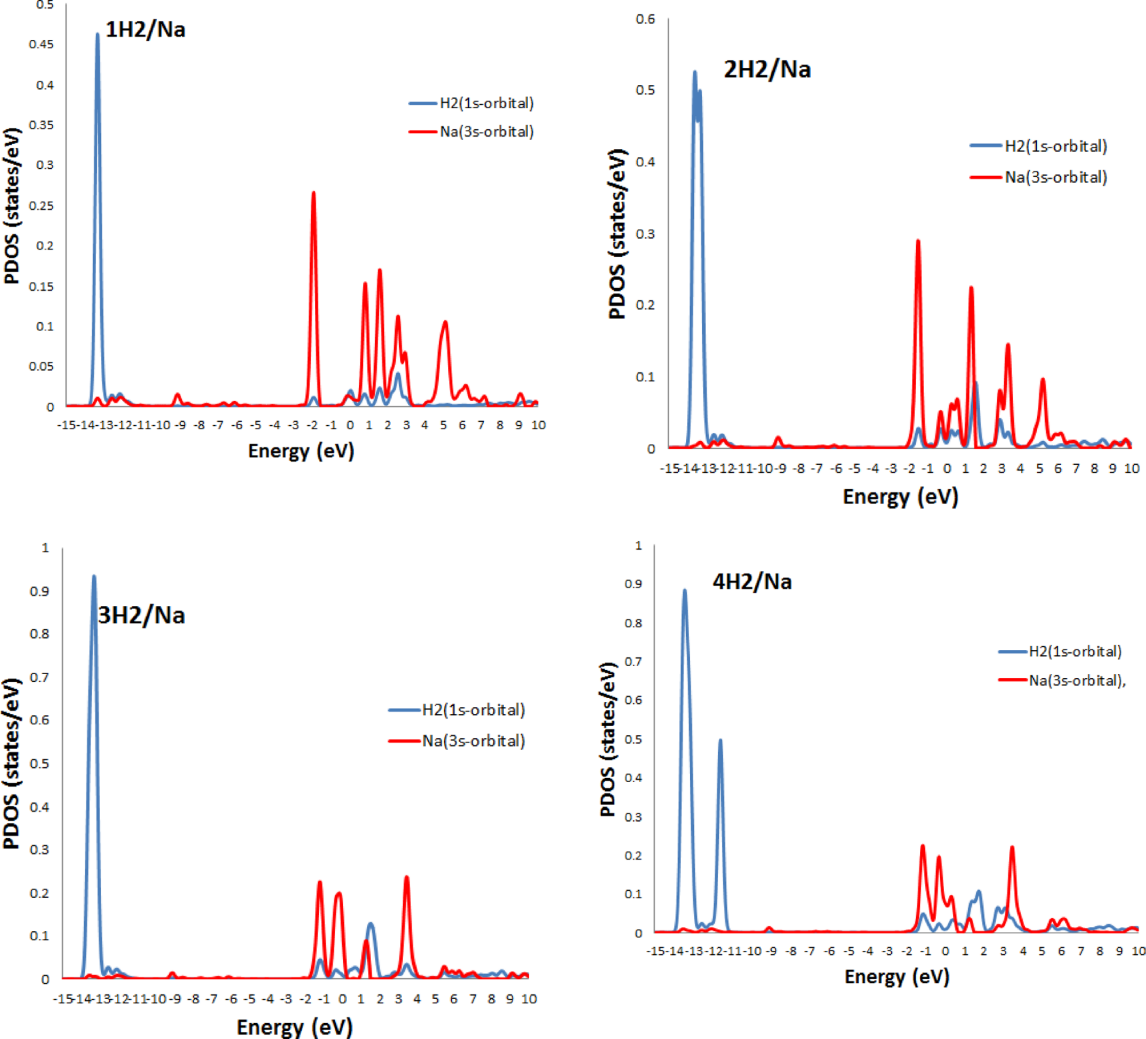
PDOS of H_2_ molecules on Na/BC_4_N monolayer.

The optimized structures of H_2_ adsorbed on Li/BC_4_N and Na/BC_4_N systems are illustrated as seen Figs. 4 and 5. The Cartesian coordinates for H_2_ adsorbed on the Li/BC_4_N and Na/BC_4_N are shown in Table S1 and S2. The PDOS of H_2_ molecules on Li/BC_4_N and Na/BC_4_N are seen in Figs 6 and 7. During the optimization of the first molecule of H_2_ on Li/BC_4_N and Na/BC_4_N systems, the H_2_ adsorbed on Li, and Na with tilted orientation on a lithium and sodium atom and This initial configuration establishes a specific binding geometry. By analyzing the results that collected in Tables 2 and 3, we can find out that the adsorption energy of −0.12 eV, and, the distance of d_Li−H_ is 2.09 Å in H_2_/Li/BC_4_N, and the distance of d_Na−H_ is 2.49 Å in H_2_/Na/BC_4_N. For each configuration the H-H bond lengths of H_2_ molecules are 0.75 Å, which are consistent with the calculated free H_2_ molecule (0.750 Å), indicating that the adsorbed H_2_ molecule is in the non-dissociative form. To further validate the physisorptive nature and reliability of our H_2_ adsorption data, we re-examined the H-H bond length before and after adsorption. In our calculations (GGA-PBE + D3), the free H_2_ bond length is 0.750 Å, and upon adsorption it remains 0.750 Å. This negligible change is fully consistent with the findings of Dange et al.^38^, who report that charge polarization driven H_2_ physisorption on NLi_4_ decorated boron phosphide biphenylene induces an H-H elongation of only ≈ 0.003 Å. Therefore, the unchanged H-H distance in our system confirms that we are describing a true physisorption process with no spurious chemisorption artifacts. The adsorption of another hydrogen molecule on the opposite side of the Li and Na decorated BC_4_N system. The adsorption energy increases to −0.27 eV for Li/BC_4_N and − 0.19 eV for Na/BC_4_N surface, suggesting a stronger interaction. The charge on Li is 0.85e, on Na is 0.93e and the charges of H_2_ on Li and Na are 0e,0.04e, 0.04e,−0.03e respectively. This indicates potential charge redistribution within the system, impacting bonding and stability. The high dipole moment (19.86 Debye and 19.60 Debye) for the first adsorbed H_2_ molecule signifies significant polarization, implying the H_2_ molecule is distorted upon interaction with the Li and Na atom. The rippling of the BC_4_N surface evident in Figs. 4 and 5 arises directly from the physisorption of H_2_, the weak van der Waals interaction and slight charge polarization at the adsorption site pull the nearest B and N atoms out of the plane, producing the observed distortion. To ensure that these distorted geometries are true minima, we have performed full vibrational frequency calculations on each H_2_ adsorbed configuration with no imaginary modes, confirming that the rippled structures are stable adsorption complexes rather than transition state artifacts .

**Fig. 8:**
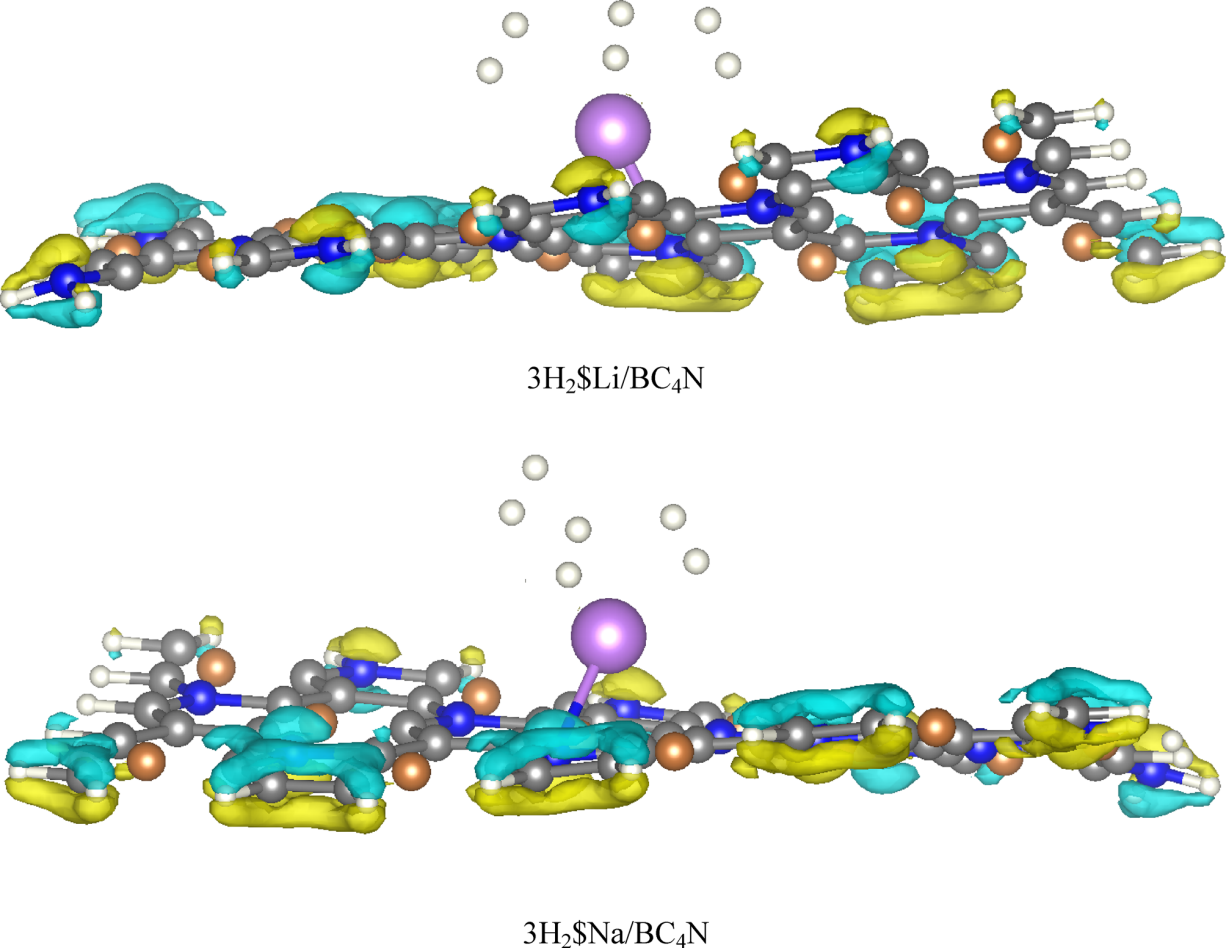
The Charge density difference of 3H_2_ on Li, and Na decorated BC_4_N.

With an increasing number of hydrogen molecules, a noteworthy trend is observed. The adsorption energy continues to increase that the two, Three, and four H_2_ molecules on Li/BC_4_N surface are − 0.19 eV, −0.25 eV, and − 0.27 eV respectively. Binding strength increases with the number of adsorbed H_2_ molecules, reaching a maximum with four molecules (−0.27 eV). This suggests cooperative effects enhance binding as more H_2_ are present. As shown in Table 2, the distance between Li decorated BC_4_N surface are 2.17Å, 2.18 Å for the two H_2_ molecules, 2.24Å, 2.30Å, 2.26Å for the three H_2_ molecules on Li/BC4N surface, but when the four H_2_ molecules we found that one H_2_ is far away from the Li decorated BC_4_N surface, and the charges obtained from NBO of Li atom is decreased by increasing H_2_ molecules adsorbed as seen in Table 2 implying a cumulative effect of hydrogen adsorption. Simultaneously, the charge on lithium decreases. This suggests a dynamic charge transfer during the adsorption process. Li-H distances and charge transfer to H_2_ molecules slightly increase with more H_2_ adsorption. This indicates stronger electrostatic interactions between Li and H_2_ molecules. As shown in Fig. 8, regions of electron accumulation (yellow) appear primarily around the Li and Na sites and the nearest H_2_ molecules, while corresponding depletion (cyan) is observed on adjacent B and N atoms. This polarization pattern confirms that H_2_ adsorption is driven by charge–induced dipole interactions at the metal centers, reinforcing the physisorptive mechanism without covalent bond formation. The increasing adsorption energy with multiple H_2_ molecules and efficient utilization of Li sites are encouraging characteristics. As seen in Table 3, we observed in Na/BC_4_N surface that the recorded adsorption energies of two, three, and four H_2_ molecules are − 0.19 eV, −0.25 eV, and − 0.23 eV respectively. Also as seen in Table 3, the distance between Na-H are 2.52Å, 2.50Å for two H_2_ molecules, 2.55Å, 2.56Å, 2.52Å, and one H_2_ molecules are far away when adsorbing four H_2_ molecules. The distances of H-H bond are in range 0.74Å−0.75Å. To account for dispersion interaction, DFT-D3 method, the adsorption energy of hydrogen molecules on Li and Na decorated BC_4_N surface as listed in Tables 2 and 3 are lower when using the Grimme D3 dispersion correction. D3 adds an attractive dispersion term to the potential energy surface, accounting for the weak interaction between hydrogen molecules with the Li and Na decorated BC_4_N surface. This stronger attractive interaction leads to a lower (more negative) adsorption energy, indicating a more stable binding.

**Table 2:** Structural and energetic properties for the hydrogen molecule adsorbed on Li/BC4N on one and two sided. The smaller Li-H distance (d_Li−H_), the bond length of H-H, charge transfer (Q), dipole moment (µ).

System	d_Li−H_(Å)	d_H−H_(Å)	E_ads_(eV)	Q^Li^(e)	Q^H^(e)	µ (Debye)
1H_2_$Li/BC_4_N	2.09	0.75	−0.12 (−0.21)	0.82	0.03, 0.01	19.86
2H_2_$Li/BC_4_N	2.18, 2.17	0.75	−0.19 (−0.36)	0.74	0.02, 0.03, 0.04, 0.01	12.87
3H_2_$Li/BC_4_N	2.24, 2.30, 2.26	0.75	−0.25 (−0.48)	0.66	0.04, 0.01, 0.01, 0.04, 0.06, −0.00	12.91
4H_2_$Li/BC_4_N	6.04, 2.24, 2.31, 2.26	0.74, 0.75	−0.27 (−0.59)	0.66	0.04, 0.01, 0.01, 0.04, 0.06, −0.00, 0.01, −0.01	13.57
2H_2_$Li_2_/BC_4_N	2.10, 2.10	0.75	−0.27	0.85, 0.85	0.04, 0.00, 0.04, 0.00	8.00
4H_2_$Li_2_/BC_4_N	2.18, 2.15, 2.18, 2.15	0.75	−0.45	0.77, 0.77	0.01, 0.04, 0.05, 0.00, 0.01, 0.04, 0.04, 0.00	8.29
6H_2_$Li_2_/BC_4_N	2.25, 2.24, 2.22, 2.23, 2.21, 2.28	0.75	−0.59	0.68, 0.68	0.05, 0.00, 0.01, 0.04, 0.05, 0.01, 0.01, 0.04, 0.05, 0.01, 0.00, 0.05	8.12
8H_2_$Li_2_/BC_4_N	6.53, 2.25, 2.24, 2.22, 4.60, 2.22, 2.20, 2.28	0.74, 0.75	−0.61	0.68, 0.68	0.05, 0.00, 0.01, 0.04, 0.05, 0.01, 0.01, 0.04, 0.05, 0.01, 0.00, 0.05, 0.01, −0.00, 0.01, −0.02	8.28

**Table 3:** Structural and energetic properties for the hydrogen molecule adsorbed on Na/BC_4_N on one and two sided. The smaller Na-H distance (d_Na−H_), the bond length of H-H, charge transfer (Q), dipole moment (µ).

System	d_Na−H_(Å)	d_H−H_(Å)	E_ads_(eV)	Q^Na^(e)	Q^H^(e)	µ (Debye)
1H_2_$Na/BC_4_N	2.49	0.75	−0.12 (−0.14)	0.92	0.05,−0.03	19.60
2H_2_$Na/BC_4_N	2.52,2.50	0.75	−0.19 (−0.33)	0.87	−0.01, 0.03, 0.05, −0.03	19.06
3H_2_$Na/BC_4_N	2.55,2.56,2.52	0.75	−0.25 (−0.41)	0.81	0.04,−0.01,0.00, 0.03,0.05,−0.02	12.90
4H_2_$Na/BC_4_N	2.53,2.53,2.52,6.18	0.74, 0.75	−0.23 (−0.53)	0.81	0.04,−0.01, 0.00, 0.03,0.05, −0.02, 0.01, −0.01	19.29
2H_2_$Na_2_/BC_4_N	2.48,2.52	0.75	−0.19	0.93, 0.93	0.04,−0.03, 0.04,−0.03	7.20
4H_2_$Na_2_/BC_4_N	2.52,2.49,2.50,2.53	0.75	−0.35	0.89, 0.89	−0.01,0.03,0.04, −0.02, −0.01,0.03, 0.04,−0.02	7.41
6H_2_$Na_2_/BC_4_N	2.54,2.54,2.54, 2.52,2.55,2.54	0.75	−0.52	0.83, 0.83	0.04,−0.02,−0.01, 0.04,0.04,−0.02, −0.01,0.03,0.04, −0.02, −0.02, 0.05	7.25
8H_2_$Na_2_/BC_4_N	2.54,2.54,2.54,6.39, 2.57,2.59,2.55,2.85	0.74, 0.75	−0.56	0.83, 0.78	0.04,−0.02,−0.01, 0.04,0.04,−0.02, −0.01, 0.03,0.05, −0.02,−0.02,0.05, 0.01,0.01,0.02, −0.02	7.29

As seen in Table 2 dipole moment of Li-decorated graphene increases with the number of adsorbed H_2_ molecules (from 12.87 Debye for two H_2_ to 13.57 Debye for four H_2_) is an interesting phenomenon with potential implications for hydrogen storage and other applications. When H_2_ interacts with Li or Na on BC4N surface, charge transfer occurs. H_2_ slightly donates some electron density to the Li, and Na atom, creating a small individual dipole moment around each H_2_-Li, and H_2_-Na pair. When the H_2_ adsorbs on the opposite side of Li or Na atom decorated BC_4_N surface, its individual dipole moment might partially cancel out the dipole moment created by the H_2_ molecules that adsorbed on one side. This could occur if the two H_2_ molecules are oriented in a specific way, with their positive and negative ends pointing towards each other.

The partial density of states (PDOS) provides valuable insights into the electronic interactions between adsorbed H_2_ molecules and the Li- and Na- decorated BC_4_N surface, as shown in Figs. 6 and 7. The third hydrogen molecule’s adsorption energy on Li, Na atom decorated BC_4_N surface is slightly higher than the initial two, as depicted in Tables 2 and 3. This behavior is elucidated by examining the Partial Density of States (PDOS). The energy bands associated with absorbed H_2_ molecules exhibit splitting below the Fermi.

level when increasing hydrogen molecules at $\:\sim\:$−14 eV. This observation implies interplay between the absorbed hydrogen molecules, indicating a potential interaction between them and proposed to enhance the adsorption strength of the third hydrogen molecule, resulting in the elevated adsorption energy. Additionally, the overlap of H_2_ orbitals with Li-2s in specific energy intervals from − 1 eV to 5 eV, and from − 2 eV to 4 eV for the overlap H_2_−1s orbitals with Na-3s orbitals signifies electrostatic interactions.

In determining the maximum hydrogen storage gravimetric density (GD) of Li and Na decorated BC_4_N monolayers, a systematic addition of both metal atoms and H_2_ molecules is required. For the Li and Na atom decorated BC_4_N monolayer, it was observed that three H_2_ molecules on the upper layer represent the maximum capacity, as adding an extra H_2_ molecule is too distant from the decorated atom. In the case of decorating both sides with Li and Na on the BC4N monolayer, the maximum storage capacity increased to six H_2_ molecules. The gravimetric capacity is calculated by the following equation:

1 $$\:\text{G}\text{D}\:=\frac{n{m}_{H2}}{{m}_{adsorbent+\:}n{m}_{H2}}\:\times\:100\%$$

Where$\:\:n{m}_{H2}$, and $\:{m}_{adsorbent\:}$are the mass of H_2_ molecules and mass of adsorbent respectively. n is the number of hydrogen molecules. By using the above equation, Li/BC_4_N storage 12.2$\:\%$ of hydrogen molecules, and Na/BC_4_N has 9.2$\:\%\:$storage capacities.

Desorption temperature represents the temperature at which hydrogen molecules, previously adsorbed onto a storage material, are released from the material, making the stored hydrogen available for use. The desorption temperature is a critical parameter in hydrogen storage systems, influencing the efficiency and practicality of the release process. Understanding and optimizing desorption temperatures is essential for designing.

effective storage materials. The hydrogen desorption temperature (TD) in a hydrogen storage system can be approximated using the Van’t Hoff formula^39^:

2 $$\:{T}_{D}=-\frac{{E}_{ads}}{{K}_{B}}{\left(\frac{\varDelta\:S}{R}-ln\frac{p}{{p}^{^\circ\:}}\right)}^{-1}$$

Where $$\:{K}_{B}\:$$is Boltzmann constant, $$\:\varDelta\:S$$ is the entropy change from gas to liquid phase of H2 molecule, R is the universal gas constant, $$\:{p}^{^\circ\:}$$ and p are standard atmospheric pressure (1 atm) and equilibrium pressure respectively^40–42^. The $$\:{T}_{D}\:$$ for 6H_2_$2Li/BC_4_N, and 6H_2_$2Na/BC_4_N are 745 K and 664 K respectively. The connection between the desorption temperature of H_2_ molecule and the applied pressure, noting that a decrease in pressure leads to a lower T_D_. This trend is explained by the proportional association between the chemical potential of H_2_ gas and pressure. Utilizing this insight, it becomes possible in practical scenarios to achieve the release of all stored H_2_ molecules at temperatures considered acceptable by effectively modulating the pressure.

### AIMD *computations*

**Fig. 9:**
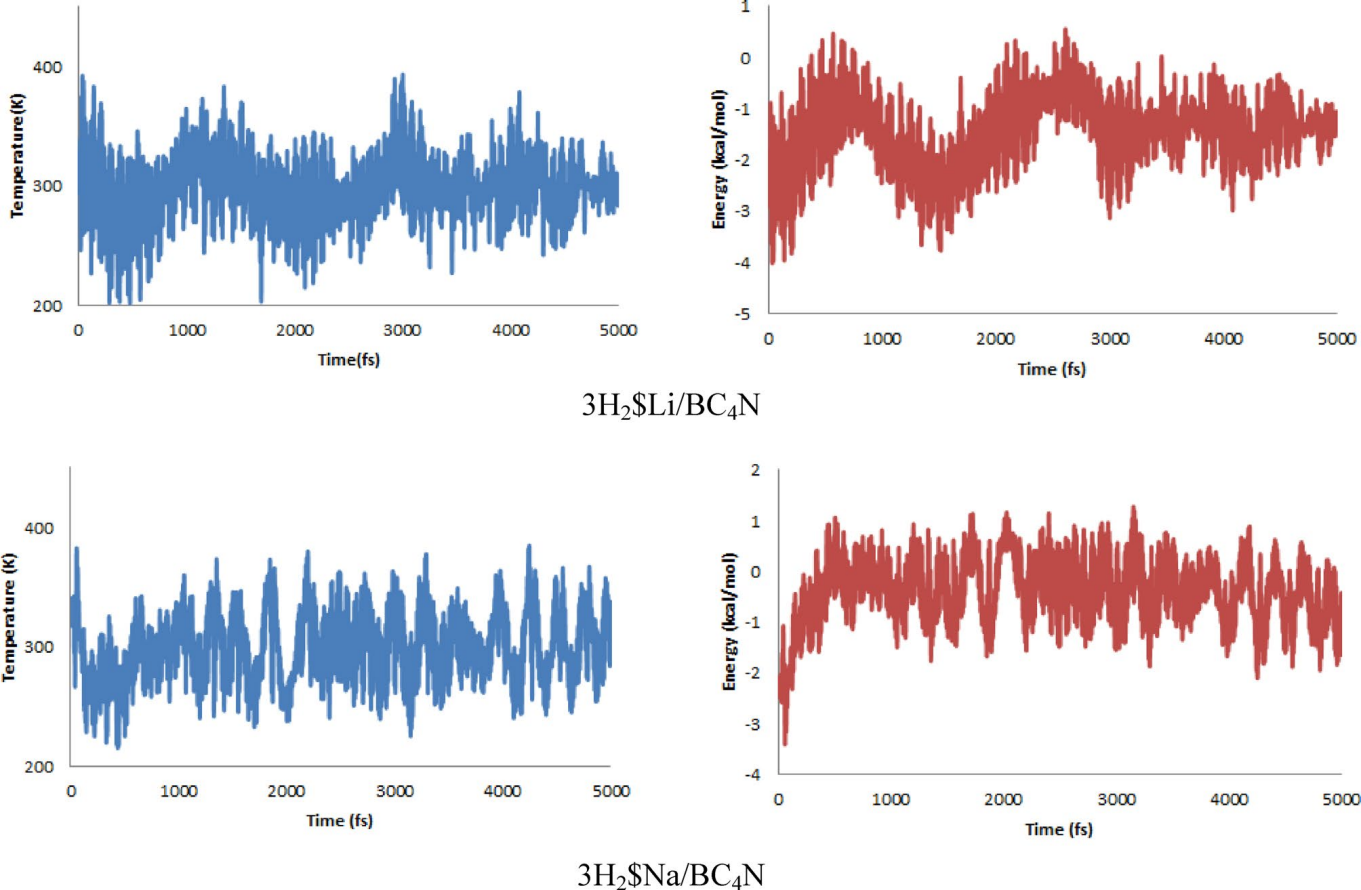
Temperature and Energy profiles from AIMD simulations of 3H2 on Li, and Na decorated BC4N.

To assess the thermal and structural stability of the H_2_ adsorbed complexes at finite temperature, we performed ab initio molecular dynamics (AIMD) simulations (300 K, and Berendsen thermostat) on the 3H_2_$Li/BC_4_N and 3H_2_$Na/BC_4_N systems. Figure 9 shows that the temperature remains well controlled around 300 K, and Fig. 9 illustrates that the total energies of both systems fluctuate within a narrow window of ± 0.02 eV, with no drift towards positive energies. Visual inspection of snapshots throughout the 10 ps trajectory revealed no bond breaking or surface reconstructions. These results confirm that the Li- and Na- decorated BC_4_N monolayers can stably host up to three H_2_ molecules at ambient conditions, with both structural integrity and adsorption reversibility maintained.

## Conclusion

Co-doped graphene (BC_4_N) exhibits favorable characteristics for hydrogen storage applications. The co-doping introduces structural ripples, enhancing binding energies for Li and Na atoms, preventing clustering. Results reveal that charge transfer from Li and Na to BC_4_N surface. H_2_ adsorption on Li/BC_4_N and Na/BC_4_N systems shows non-dissociative behavior. The calculated hydrogen storage gravimetric density suggests significant storage capacities for both Li/BC_4_N and Na/BC_4_N. Desorption temperatures are determined, providing insights for practical applications. Overall, co-doped graphene BC_4_N emerges as a promising material for efficient and practical hydrogen storage.

### Computational details

The study involves a comprehensive geometry optimization of various structures, conducted using the B3LYP/6–31 g(d, p) level of theory. The calculations are executed through the Gaussian 09 code^43^. The study also incorporates the dispersion correction method known as DFT-D3, based on the Grimme scheme^44^. This method is employed to accurately account for weak van der Waals interactions occurring between H_2_ molecules and the substrates under consideration.

The focus of the investigation is on co-doped graphene, where non-bonded B and N atoms (BC_4_N) are present, and these structures are terminated with hydrogen atoms. The binding energy (E_b_) between lithium and sodium decorated BC4N monolayers calculated are shown as following:-

3 $$\:{E}_{b}={E}_{M/BC4N}-\left({E}_{BC4N}+{E}_{M}\right)$$

Where $\:{\text{E}}_{\text{M}/\text{B}\text{C}4\text{N}}$, $\:{\text{E}}_{\text{B}\text{C}4\text{N}},\:\:{\text{a}\text{n}\text{d}\:\text{E}}_{\text{M}}$ are the total energies of Li, Na decorated BC_4_N, energy of BC_4_N, and energy of the isolated decorated-atom respectively.

The calculation of hydrogen adsorption energy (E_ads_) on a Li, and Na over the stable position was calculated using equation:

4 $$\:{E}_{ads}={E}_{H2\$M/BC4N}-({E}_{M/BC4N}+{E}_{H2})$$

Where $\:{E}_{H2\$M/BC4N},{E}_{M/BC4N},\:\:\text{a}\text{n}\text{d}\:{E}_{H2}$ are referred to the H_2_ molecules adsorbed on decorated atom, and the energy of the free H_2_ molecules respectively.

Charge density difference (CDD) plots were utilized to investigate the electron rearrangement accompanying H_2_ adsorption. The CDD was computed as^38^.

5 $$\:{\Delta\:}\rho\:=\:{\rho\:}_{3\text{H}2\text{\$}\text{M}/\text{B}\text{C}4\text{N}}-{\rho\:}_{\text{M}/\text{B}\text{C}4\text{N}}-\:{\rho\:}_{3\text{H}2}$$

Where $\:{\rho\:}_{3\text{H}2\text{\$}\text{M}/\text{B}\text{C}4\text{N}}$ is the total charge density of the Li, and Na decorated BC_4_N sheet with three adsorbed H_2_ molecules, and $\:{\rho\:}_{\text{M}/\text{B}\text{C}4\text{N}}$ and $\:{\rho\:}_{3\text{H}2}$are those of the Li, and Na decorated BC4N sheet and gas phase H_2_ cluster, respectively.

## Supplementary Information

Below is the link to the electronic supplementary material.

Supplementary Material 1

## Data Availability

No datasets were generated or analysed during the current study.
