# Supplementary material for: DFT investigation of efficient hydrogen storage utilizing Li and Na decorated co-doped graphene (B/N)
Source: Sci Rep. 2025 Aug 19;15:30371. doi: 10.1038/s41598-025-14088-8 (PMC12365210; doi:10.1038/s41598-025-14088-8)
Supplement: Supplementary file 1 — Supplementary Material 1 [file 41598_2025_14088_MOESM1_ESM.docx]

Table S1: the Cartesian coordinated for the Li/BC_4_N, and H_2_ interactions on Li/BC_4_N

Li/BC_4_N

C 8.04677200 -2.36579700 -0.25310600

C 8.21631400 -4.96054200 -0.70409700

C 7.95939700 2.39603100 0.48330400

C 7.85408900 0.03895200 0.12671900

C 5.81581900 -1.28762200 -0.13474000

C 7.24498900 -1.22465700 -0.08921500

C 5.84147400 -3.67593200 -0.52146600

C 3.76568800 -5.01635300 -0.71889500

C 5.12517200 -4.88735900 -0.72728900

C 8.14070100 4.98509200 0.85749900

C 5.77562200 3.70655500 0.51187800

C 5.08145800 2.46667900 0.31821500

C 5.82159600 1.26753200 0.21331600

C 3.57243800 0.02000600 -0.02931800

C 2.93235700 -1.26374800 -0.16315000

C 3.71213400 -2.46085700 -0.32004200

C 1.43753700 -3.73790500 -0.35138400

C 0.72156700 -4.96640200 -0.30667800

C 3.72082000 4.98818800 0.48384800

C 3.64617000 2.44364700 0.22055200

C 1.50239800 1.26893300 0.00593300

C 1.49499400 -1.32386100 -0.14625600

C -0.66233100 -2.51046600 -0.05066100

C -0.61618200 -5.08782400 -0.07043600

C 1.38429700 3.72237300 0.09926500

C -0.71191900 2.42884500 -0.08665800

C 0.72387000 2.46195100 0.00949300

C -0.77666100 -0.00660300 -0.07734600

C -2.87356800 -1.34842000 0.07724200

C -1.44356500 -1.28475300 -0.02936500

C -2.92649300 -3.76416100 0.29594800

C -4.98603800 -5.05779500 0.71231300

C -3.62494800 -4.96229700 0.53843100

C -0.65893400 4.99426200 -0.11134200

C -2.97915400 3.68613300 -0.33745100

C -3.63974500 2.43561300 -0.25496300

C -2.85110600 1.24176100 -0.12704700

C -5.11520200 -0.02799200 0.00866500

C -5.75111200 -1.26613900 0.20473500

C -5.00779000 -2.50448300 0.33857500

C -7.32718600 -3.72395000 0.69709200

C -8.18010100 -4.75550800 0.94084300

C -5.01655100 4.94278600 -0.63320100

C -5.08045300 2.39486900 -0.30454700

C -7.20280000 1.23273200 -0.16249500

C -7.18961100 -1.31317100 0.27192700

C -7.36665700 3.66636700 -0.59462300

C -7.92901900 2.41766600 -0.37867600

B 7.39473600 -3.69683700 -0.49976300

B 2.95881700 -3.75725700 -0.48260000

B -1.41406100 -3.80830600 0.07710600

B -5.77073300 -3.79271400 0.59283600

N 5.13103500 -2.48050400 -0.32953000

N 0.76607000 -2.53165400 -0.20558800

N -3.60378800 -2.54436000 0.23395900

N -7.86865200 -2.46126200 0.50303400

B 5.07235300 -0.00606200 0.01781500

B 0.74338800 -0.01550700 -0.10092500

B -3.60702000 -0.04482400 -0.01542100

B -7.97794800 -0.04537700 0.06851800

N 7.22438000 1.24213900 0.27786200

N 2.90982200 1.24236000 0.06462900

N -1.45308700 1.22468500 -0.09639200

N -5.80149500 1.19634700 -0.15272600

B 7.32522000 3.74027700 0.62914900

B 2.90089100 3.75719500 0.27362700

B -1.46578700 3.73648300 -0.18534500

B -5.83879200 3.69741700 -0.52449200

N 5.06377900 4.85281200 0.58774100

N 0.67603400 4.87461300 0.03499200

N -3.68277300 4.83906100 -0.52110300

N -8.16066900 4.75847100 -0.79784600

H 7.83553500 -5.96367500 -0.89024900

H 9.30563400 -4.92516700 -0.67613800

H 9.12680000 -2.22230600 -0.20027400

H 8.93472700 0.09334100 0.17552500

H 9.02875500 2.20566300 0.51688200

H 7.75296900 5.99950700 0.97910500

H 9.22730200 4.95715700 0.92475600

H 5.62952000 5.68243100 0.73337800

H 3.39037700 6.02039400 0.58204800

H 1.23330000 5.71903300 0.09728600

H -0.99478600 6.02828100 -0.13924100

H -3.12130900 5.68030300 -0.58123200

H -5.36488200 5.96249700 -0.77729000

H -9.14954200 4.64111500 -0.96278100

H -7.75904400 5.61508400 -1.14067100

H -9.01088100 2.29678100 -0.37231400

H -9.17518700 -0.04381300 0.08931000

H -8.87877000 -2.36354200 0.53206400

H -9.25884500 -4.62066000 0.99101600

H -7.80404900 -5.75867000 1.09378800

H -5.40457900 -6.04454600 0.90319300

H -3.00434400 -5.85168400 0.58769700

H -1.02732500 -6.09443100 -0.04175800

H 1.33576200 -5.84936300 -0.44921600

H 5.75679400 -5.75474800 -0.89054200

H 3.35936000 -6.01150800 -0.88872000

Li 0.10117300 -1.41437400 1.65684000

1H_2_$Li/BC_4_N

C -8.04832900 -2.35398700 -0.25915100

C -8.21816500 -4.95073400 -0.69821600

C -7.96059800 2.41000800 0.46023900

C -7.85532800 0.05193500 0.11159300

C -5.81709800 -1.27558700 -0.14492100

C -7.24630800 -1.21239900 -0.09967300

C -5.84326100 -3.66524600 -0.52223800

C -3.76798100 -5.00665300 -0.71567100

C -5.12738200 -4.87768000 -0.72329200

C -8.14194600 5.00023900 0.82611600

C -5.77666500 3.71995700 0.48830900

C -5.08238700 2.47946800 0.29879900

C -5.82261500 1.28023700 0.19606300

C -3.57309300 0.03171200 -0.04206100

C -2.93295000 -1.25226100 -0.17477300

C -3.71349300 -2.44940200 -0.32763400

C -1.43942700 -3.72647400 -0.35992400

C -0.72391000 -4.95492100 -0.31960700

C -3.72170000 5.00101200 0.46136000

C -3.64699600 2.45574500 0.20331900

C -1.50280700 1.28024600 -0.00676700

C -1.49533400 -1.31235200 -0.15994000

C 0.66245200 -2.49857200 -0.07785600

C 0.61647000 -5.07641800 -0.09822900

C -1.38494200 3.73385500 0.08587600

C 0.71150900 2.44018900 -0.09495800

C -0.72442700 2.47319900 -0.00210600

C 0.77669200 0.00487800 -0.08879900

C 2.87426400 -1.33749600 0.05295100

C 1.44363700 -1.27353600 -0.04990800

C 2.92869400 -3.75489400 0.25096000

C 4.99015300 -5.05265000 0.64465800

C 3.62820800 -4.95520100 0.47915400

C 0.65860900 5.00600400 -0.11734800

C 2.97852800 3.69881100 -0.33834700

C 3.64003500 2.44907400 -0.25301800

C 2.85151100 1.25411600 -0.13243700

C 5.11628100 -0.01578700 -0.00685200

C 5.75296800 -1.25594200 0.17335600

C 5.01005300 -2.49582800 0.29599300

C 7.33128600 -3.71990800 0.63037600

C 8.18507800 -4.75429700 0.85727200

C 5.01531900 4.95889800 -0.62137200

C 5.08105000 2.41052700 -0.29530000

C 7.20362700 1.24750500 -0.16436700

C 7.19162000 -1.30409300 0.23502400

C 7.36856500 3.69036500 -0.54106000

C 7.93029300 2.43606400 -0.35308800

B -7.39649100 -3.68611400 -0.49992200

B -2.96079500 -3.74641200 -0.48606700

B 1.41533100 -3.79700900 0.04070500

B 5.77421400 -3.78662000 0.53331000

N -5.13248100 -2.46915400 -0.33570100

N -0.76612500 -2.51957400 -0.22083500

N 3.60574500 -2.53452800 0.19675100

N 7.87179300 -2.45479900 0.44889400

B -5.07321800 0.00610400 0.00442600

B -0.74351200 -0.00430700 -0.11271100

B 3.60772600 -0.03285400 -0.02925500

B 7.97934100 -0.03377800 0.04727300

N -7.22543200 1.25537700 0.25928400

N -2.91042500 1.25404700 0.05038200

N 1.45309900 1.23626100 -0.10476100

N 5.80221800 1.21065500 -0.15533100

B -7.32641400 3.75452600 0.60289800

B -2.90182400 3.76920700 0.25577100

B 1.46489900 3.74809100 -0.19109500

B 5.83918600 3.71665300 -0.49529900

N -5.06486500 4.86630600 0.56270900

N -0.67682800 4.88618600 0.02458700

N 3.68059000 4.85230600 -0.52301700

N 8.16179000 4.77824000 -0.74957900

H -7.83754900 -5.95477700 -0.87980300

H -9.30748900 -4.91515400 -0.67021400

H -9.12833000 -2.20997400 -0.20700300

H -8.93598300 0.10668600 0.15956100

H -9.03001400 2.21981000 0.49300800

H -7.75426700 6.01502100 0.94488900

H -9.22866600 4.97273000 0.89174000

H -5.63079000 5.69636900 0.70501700

H -3.39120200 6.03341500 0.55744400

H -1.23437900 5.73042800 0.08642500

H 0.99496400 6.03995200 -0.14140900

H 3.11683900 5.69060100 -0.60040500

H 5.36063600 5.97702200 -0.78365700

H 9.16473100 4.71212100 -0.66497800

H 7.77218200 5.70497800 -0.76720200

H 9.01213200 2.31539300 -0.34474500

H 9.17662600 -0.03199100 0.06714900

H 8.88200400 -2.35731200 0.47449500

H 9.26411600 -4.62026800 0.90330600

H 7.80965800 -5.75921900 0.99986300

H 5.40960100 -6.04125400 0.82365100

H 3.00805100 -5.84514600 0.52283700

H 1.02728000 -6.08323200 -0.07246300

H -1.33970300 -5.83791200 -0.45472900

H -5.75924100 -5.74568500 -0.88229000

H -3.36205700 -6.00262200 -0.88169500

Li -0.10175700 -1.41190700 1.64925300

H 0.17966700 -2.98599100 3.00052500

H -0.19787900 -2.54532800 3.47403700

2H_2_$Li/BC_4_N

C -8.04248200 -2.34385700 -0.31612800

C -8.23912000 -4.91377400 -0.77531400

C -7.96226000 2.40791100 0.44682100

C -7.85464300 0.05397400 0.07645000

C -5.81108000 -1.27500700 -0.17689600

C -7.23703000 -1.20398800 -0.14008700

C -5.83468500 -3.66305200 -0.57199300

C -3.76109300 -5.00285500 -0.75204100

C -5.12263400 -4.87320400 -0.77227700

C -8.14185600 4.99616100 0.83629400

C -5.77726100 3.71196600 0.49627100

C -5.08075400 2.47522000 0.29957600

C -5.82163800 1.27545200 0.18345200

C -3.56673600 0.02900300 -0.05326500

C -2.92749900 -1.25122300 -0.19059300

C -3.70898500 -2.45064800 -0.35286400

C -1.43235800 -3.72662400 -0.37227600

C -0.71872800 -4.95344300 -0.32085500

C -3.72447200 4.99463000 0.48529000

C -3.64518100 2.45219300 0.20838600

C -1.50058800 1.27879600 -0.00769400

C -1.49027800 -1.31224000 -0.17363400

C 0.66626200 -2.49754500 -0.08745300

C 0.62226500 -5.07311000 -0.09413400

C -1.38465100 3.73200400 0.10277200

C 0.71254200 2.44187000 -0.09379700

C -0.72213900 2.47446200 0.00419400

C 0.77983700 0.00406400 -0.09993600

C 2.87866000 -1.33476100 0.04420600

C 1.44704100 -1.27117100 -0.05981000

C 2.93451100 -3.75112900 0.25429300

C 4.99594700 -5.04274500 0.66494600

C 3.63403500 -4.94796600 0.49225400

C 0.65556800 5.00624600 -0.09710300

C 2.97828800 3.70329800 -0.34115500

C 3.64030000 2.45084900 -0.27064200

C 2.85134200 1.25690400 -0.14841900

C 5.11769500 -0.01245900 -0.02286000

C 5.75961800 -1.25000500 0.16870900

C 5.01589400 -2.48744800 0.29867200

C 7.33795300 -3.71080800 0.65474100

C 8.18202600 -4.74882500 0.89729700

C 5.01678600 4.95737900 -0.63379000

C 5.08084300 2.40700300 -0.32568900

C 7.20744900 1.24389200 -0.19269300

C 7.19848100 -1.29509900 0.23484700

C 7.36698200 3.68065100 -0.62620800

C 7.92893500 2.43921500 -0.41381700

B -7.38751700 -3.67156900 -0.56207600

B -2.95534100 -3.74661700 -0.50965000

B 1.42265100 -3.79468400 0.03754000

B 5.78173100 -3.77861000 0.54935600

N -5.12604300 -2.47023400 -0.37171500

N -0.75948400 -2.52068400 -0.23433900

N 3.61182400 -2.52962900 0.19541100

N 7.87823900 -2.44798000 0.46396100

B -5.06833700 0.00422100 -0.01363700

B -0.73916600 -0.00628800 -0.12093900

B 3.61228300 -0.03165400 -0.04487000

B 7.98077000 -0.02768400 0.03308600

N -7.22452100 1.25486900 0.23923500

N -2.90495500 1.25103600 0.04853400

N 1.45576500 1.23636000 -0.11353300

N 5.80248300 1.20911500 -0.18082500

B -7.32663300 3.74819200 0.60476800

B -2.90043300 3.76428600 0.27163300

B 1.46701300 3.74914700 -0.18441000

B 5.84034600 3.71144200 -0.54379900

N -5.06610200 4.85952600 0.58227700

N -0.67683300 4.88652500 0.04912700

N 3.68211100 4.85570200 -0.51556800

N 8.15670400 4.79093300 -0.82314000

H -7.88004200 -5.92428400 -0.96426000

H -9.32700800 -4.85121900 -0.75229600

H -9.12270800 -2.20065900 -0.27154800

H -8.93557700 0.10615900 0.11693900

H -9.03160100 2.21685200 0.47182300

H -7.75263600 6.00863700 0.96706800

H -9.22883000 4.97000500 0.89627400

H -5.63242300 5.68852200 0.72928700

H -3.39385000 6.02614100 0.58963900

H -1.23328400 5.73092000 0.11851300

H 0.99253000 6.04010300 -0.11640300

H 3.12420100 5.69993300 -0.56223500

H 5.36752600 5.97800700 -0.76397800

H 9.13009300 4.64935300 -1.05550600

H 7.74187300 5.59538200 -1.26632100

H 9.01111400 2.31796800 -0.41322300

H 9.17928600 -0.02472100 0.05114700

H 8.88751800 -2.34872100 0.49323300

H 9.26159800 -4.62167200 0.94994500

H 7.79888000 -5.74986000 1.04640400

H 5.41535400 -6.02983000 0.85209200

H 3.01526000 -5.83904200 0.53823800

H 1.03258400 -6.07984900 -0.05871900

H -1.33338800 -5.83811000 -0.45133900

H -5.75222200 -5.74175000 -0.94193300

H -3.35434600 -5.99815100 -0.91940500

Li -0.05318100 -1.41188700 1.66774000

H 0.02192700 -0.27144700 3.60436300

H -0.24200100 0.22013200 3.10744900

H -0.31857000 -3.30545000 2.68504200

H -0.72949100 -2.89834200 3.15921100

3H_2_$Li/BC_4_N

C -8.04584900 -2.34189700 -0.25177800

C -8.24665900 -4.91927600 -0.66462000

C -7.96088200 2.42048700 0.43356500

C -7.85512500 0.06140500 0.09984100

C -5.81340700 -1.26974200 -0.15742800

C -7.23890300 -1.19912800 -0.10418300

C -5.84095100 -3.66273700 -0.51895800

C -3.76978900 -5.00222200 -0.72336000

C -5.13139700 -4.87468200 -0.71689800

C -8.13894500 5.01428100 0.78426800

C -5.77615100 3.72513800 0.44994000

C -5.08043300 2.48595800 0.26570700

C -5.82172000 1.28477300 0.17009000

C -3.56791000 0.03586500 -0.07082600

C -2.92890200 -1.24507800 -0.20628000

C -3.71274500 -2.44590900 -0.34851600

C -1.43522600 -3.71973300 -0.40833300

C -0.71802300 -4.94642200 -0.39627400

C -3.72353800 5.00712600 0.41652100

C -3.64530200 2.46146300 0.16827700

C -1.50077100 1.28524400 -0.04059600

C -1.49132000 -1.30573700 -0.20382300

C 0.66514200 -2.49090200 -0.13728000

C 0.62645000 -5.06756000 -0.19944300

C -1.38440600 3.73922100 0.05345900

C 0.71334500 2.44747700 -0.12181400

C -0.72186500 2.48081300 -0.03299800

C 0.77934700 0.00942200 -0.12907100

C 2.87771500 -1.33116700 0.00856600

C 1.44541600 -1.26686200 -0.09903200

C 2.93506600 -3.75103400 0.17401700

C 4.99443500 -5.05092600 0.56853200

C 3.63394600 -4.95292300 0.38833600

C 0.65692300 5.01228400 -0.13783200

C 2.98180500 3.70925600 -0.34290400

C 3.64299500 2.45682800 -0.26619300

C 2.85239800 1.26245400 -0.15796300

C 5.11804700 -0.00816600 -0.02069700

C 5.75877700 -1.24797700 0.16031900

C 5.01478100 -2.48779300 0.26065000

C 7.33406200 -3.71667500 0.61642600

C 8.17623200 -4.75905500 0.84724100

C 5.02378100 4.96612900 -0.59599900

C 5.08428800 2.41400200 -0.30119800

C 7.20939500 1.25144600 -0.14454100

C 7.19686700 -1.29317300 0.24431700

C 7.37431200 3.69194900 -0.55361200

C 7.93357300 2.44937600 -0.34179000

B -7.39325500 -3.67293600 -0.48485200

B -2.96093800 -3.74227200 -0.51088800

B 1.42302200 -3.79008700 -0.04029400

B 5.77946200 -3.78351000 0.49090500

N -5.13013300 -2.46689600 -0.34534900

N -0.76098400 -2.51305600 -0.27224000

N 3.61149400 -2.52872800 0.14451100

N 7.87501200 -2.44966800 0.46015200

B -5.06934900 0.01153100 -0.01814400

B -0.73953200 -0.00042500 -0.15301300

B 3.61259700 -0.02725800 -0.05792700

B 7.98055400 -0.02205700 0.07648400

N -7.22411400 1.26480400 0.23792700

N -2.90555200 1.25860800 0.01772600

N 1.45651200 1.24168000 -0.13515700

N 5.80445200 1.21540700 -0.15521400

B -7.32475100 3.76311200 0.56659400

B -2.90039200 3.77383600 0.21668400

B 1.46870300 3.75451100 -0.20872400

B 5.84649800 3.72034200 -0.49820700

N -5.06478800 4.87371500 0.51846900

N -0.67648000 4.89346700 -0.00301600

N 3.68750700 4.86278900 -0.50139300

N 8.16618700 4.80465000 -0.72727400

H -7.88953800 -5.93271800 -0.84111100

H -9.33414200 -4.85703700 -0.62659900

H -9.12548000 -2.19838000 -0.19543400

H -8.93561200 0.11359500 0.15065200

H -9.02988200 2.22934100 0.46961000

H -7.74952900 6.02888900 0.89647300

H -9.22548400 4.98853700 0.85180200

H -5.63053600 5.70458400 0.65688700

H -3.39245200 6.03978100 0.50715500

H -1.23356800 5.73819100 0.05625000

H 0.99392200 6.04603100 -0.16188800

H 3.12990200 5.70689300 -0.55315600

H 5.37582400 5.98764000 -0.71523900

H 9.14358300 4.66578000 -0.94394800

H 7.75839000 5.61226300 -1.17122600

H 9.01566600 2.32873300 -0.32399200

H 9.17863900 -0.01823400 0.11349700

H 8.88368100 -2.34983300 0.50470700

H 9.25491300 -4.63213300 0.91611900

H 7.79234500 -5.76336300 0.97004300

H 5.41336500 -6.04178700 0.73566700

H 3.01529000 -5.84481700 0.41143200

H 1.04040800 -6.07332600 -0.19830300

H -1.33341500 -5.82944000 -0.53352600

H -5.76295400 -5.74603700 -0.86303100

H -3.36554100 -5.99920600 -0.88610100

Li -0.05242300 -1.41729000 1.66232800

H 1.87176200 -1.34079000 2.81236000

H 1.43436600 -1.50319200 3.39558600

H -0.88032900 -0.11469600 3.49335200

H -1.05212900 0.26664200 2.87479200

H -1.39929100 -3.11980300 2.34481700

H -1.16507700 -2.85529500 3.00330800

4H_2_$Li/BC_4_N

C -8.05272000 -2.37070400 -0.22271500

C -8.24030400 -4.95006400 -0.62837200

C -7.99324200 2.39359100 0.44969600

C -7.87471000 0.03436400 0.12219300

C -5.82640400 -1.28428600 -0.14710600

C -7.25191800 -1.22259400 -0.08348800

C -5.84163000 -3.67769200 -0.50613900

C -3.76396800 -5.00344000 -0.73112700

C -5.12622600 -4.88497800 -0.71078800

C -8.18537800 4.98740300 0.79313500

C -5.81671900 3.71178200 0.44906600

C -5.11423900 2.47641100 0.26486900

C -5.84855200 1.27045300 0.17659800

C -3.58833100 0.03514500 -0.07630300

C -2.94222000 -1.24167000 -0.21659100

C -3.71983000 -2.44716600 -0.35410900

C -1.43497100 -3.70612900 -0.43844400

C -0.71036500 -4.92838800 -0.44132400

C -3.77251400 5.00641900 0.40083200

C -3.67949800 2.46044100 0.16012300

C -1.52851200 1.29689200 -0.05522600

C -1.50413200 -1.29339800 -0.22411800

C 0.66023300 -2.46576400 -0.17820600

C 0.63664700 -5.04240600 -0.25928800

C -1.42698500 3.75164900 0.03550700

C 0.67834300 2.47240500 -0.14021900

C -0.75695600 2.49709900 -0.05021200

C 0.75969700 0.03493800 -0.15418600

C 2.86731400 -1.29379500 -0.03538100

C 1.43368600 -1.23743700 -0.13552900

C 2.93986200 -3.71498900 0.10249300

C 5.01045900 -5.00849800 0.46029000

C 3.64679700 -4.91528300 0.29611300

C 0.60630800 5.03717700 -0.15549900

C 2.93848200 3.74901200 -0.35598600

C 3.60863900 2.50211500 -0.26931900

C 2.82544500 1.30162300 -0.17211700

C 5.09968200 0.04388100 -0.05222500

C 5.74915900 -1.19530000 0.10065100

C 5.01312400 -2.44103200 0.18546800

C 7.34389900 -3.66372200 0.49117500

C 8.19462700 -4.70619700 0.68665600

C 4.97206400 5.02246100 -0.58910400

C 5.05057300 2.47095400 -0.28931500

C 7.18298900 1.31910300 -0.15445400

C 7.18822500 -1.23409200 0.17091800

C 7.33390000 3.77616000 -0.45619100

C 7.89996300 2.52839900 -0.29805000

B -7.39346500 -3.69793400 -0.45850600

B -2.96134000 -3.73835700 -0.52548700

B 1.42638400 -3.76110200 -0.09803000

B 5.78805200 -3.73596300 0.38720200

N -5.13698600 -2.47722900 -0.33920500

N -0.76686100 -2.49593800 -0.30232200

N 3.60932600 -2.48845700 0.08111900

N 7.87555800 -2.39071700 0.35402200

B -5.08933200 0.00167800 -0.01429600

B -0.75956500 0.01611700 -0.17268600

B 3.59403500 0.01571400 -0.08558700

B 7.96309400 0.04513200 0.02898200

N -7.25037200 1.24201600 0.25298900

N -2.93295000 1.26188100 0.00833300

N 1.42951200 1.27151100 -0.15439700

N 5.77825300 1.27442100 -0.16465700

B -7.36478700 3.74056100 0.57462300

B -2.94269200 3.77748300 0.20192300

B 1.42497800 3.78410600 -0.22764500

B 5.80472900 3.78738700 -0.45005100

N -5.11235600 4.86507600 0.50971600

N -0.72636400 4.91025300 -0.02143500

N 3.63520600 4.90689200 -0.52149600

N 8.11956900 4.89065200 -0.63430500

H -7.87823700 -5.96168000 -0.80522600

H -9.32780200 -4.89462400 -0.58140100

H -9.13277100 -2.23394600 -0.15833800

H -8.95513400 0.07984100 0.18020400

H -9.06077200 2.19582300 0.49269900

H -7.80148300 6.00469900 0.89996800

H -9.27133700 4.95529900 0.86693500

H -5.68272200 5.69285600 0.64769500

H -3.44753700 6.04153600 0.48544100

H -1.28857800 5.75146900 0.03916600

H 0.93787600 6.07273900 -0.17544200

H 3.06934800 5.74237400 -0.61079900

H 5.31461500 6.04188500 -0.74835600

H 9.10506200 4.82576200 -0.42084900

H 7.71531300 5.79683300 -0.46052700

H 8.98267500 2.41452400 -0.27546700

H 9.16125000 0.05583100 0.06172900

H 8.88407300 -2.28566700 0.38883600

H 9.27355700 -4.57493800 0.74183900

H 7.81783800 -5.71494100 0.79392900

H 5.43632900 -5.99932600 0.60884800

H 3.03352500 -5.81085000 0.31458600

H 1.05663500 -6.04558000 -0.26937300

H -1.32155900 -5.81433300 -0.57809500

H -5.75342200 -5.76048500 -0.85069400

H -3.35499600 -5.99775400 -0.89814100

Li -0.05338500 -1.40736500 1.63314900

H 1.86674700 -1.29604200 2.77325500

H 1.43220400 -1.45678700 3.35910400

H -0.91328200 -0.12068800 3.47251900

H -1.08381500 0.26226300 2.85464900

H -1.37774700 -3.12253700 2.32140600

H -1.13104000 -2.86513600 2.97813700

H 4.50469300 -4.88663000 3.52385200

H 4.41068200 -4.94144700 4.25980800

Table S2: the Cartesian coordinated for the Na/BC_4_N, and H_2_ interactions on Na/BC_4_N

Na/BC_4_N

C 8.04565800 -2.32866500 -0.26733800

C 8.24890100 -4.89777300 -0.73032200

C 7.95398600 2.42339700 0.49627900

C 7.85130700 0.06912300 0.12522700

C 5.81231800 -1.25842300 -0.16619600

C 7.23754300 -1.18848400 -0.10368200

C 5.84226700 -3.64463900 -0.57293400

C 3.77128300 -4.97974000 -0.80926500

C 5.13342800 -4.85207300 -0.79782200

C 8.12860600 5.01244800 0.88417900

C 5.76922800 3.72891300 0.50737000

C 5.07531500 2.49198300 0.30352400

C 5.81749600 1.29144500 0.19964900

C 3.56612800 0.04589000 -0.07297700

C 2.92796100 -1.23408500 -0.22367600

C 3.71325300 -2.43324400 -0.38495500

C 1.43548400 -3.70547100 -0.45780400

C 0.72217700 -4.93299300 -0.43648500

C 3.71699900 5.01211300 0.46271600

C 3.64142300 2.46915500 0.19366700

C 1.49842000 1.29278000 -0.02918800

C 1.49242800 -1.29589000 -0.21418300

C -0.66176200 -2.48120600 -0.14849000

C -0.61816000 -5.05668000 -0.20565200

C 1.38172000 3.74768300 0.05558500

C -0.71412800 2.45548800 -0.13501400

C 0.71939800 2.48936900 -0.03189700

C -0.77990200 0.01896000 -0.13177300

C -2.87332700 -1.32295300 0.01242500

C -1.44306000 -1.25840500 -0.09692700

C -2.92595300 -3.74091200 0.19787800

C -4.97916100 -5.03617100 0.63906200

C -3.62110900 -4.94019700 0.43829400

C -0.65781600 5.01900900 -0.16895900

C -2.98308000 3.71374400 -0.38228700

C -3.64358500 2.46144000 -0.29322900

C -2.85332000 1.26865500 -0.17248600

C -5.11557500 -0.00232800 -0.01948500

C -5.75381700 -1.24052900 0.18156000

C -5.00744200 -2.47847400 0.29291000

C -7.32138800 -3.70373400 0.68887300

C -8.16005000 -4.74310400 0.94495400

C -5.02632200 4.96500000 -0.65590700

C -5.08470200 2.41669600 -0.32853900

C -7.20842900 1.25278600 -0.15327300

C -7.19075600 -1.28604600 0.27503400

C -7.37593100 3.68725500 -0.59827400

C -7.93371800 2.44771200 -0.36732700

B 7.39507500 -3.65460900 -0.53063800

B 2.96094200 -3.72537000 -0.57118600

B -1.41593200 -3.78000300 -0.04029100

B -5.76768600 -3.77095900 0.55050300

N 5.13017100 -2.45288500 -0.37868200

N 0.76148000 -2.50154100 -0.29190300

N -3.60497900 -2.51955200 0.16366600

N -7.86559500 -2.44020700 0.51457500

B 5.06722400 0.02077500 -0.01199200

B 0.73856700 0.00673200 -0.13796500

B -3.61058300 -0.01987400 -0.06113100

B -7.97684200 -0.01820000 0.09002600

N 7.21899900 1.27009000 0.27761000

N 2.90302900 1.26677100 0.02868600

N -1.45593500 1.24797100 -0.14796200

N -5.80341900 1.21863700 -0.16680500

B 7.31671900 3.76432600 0.64114300

B 2.89570500 3.78115500 0.24179400

B -1.47009300 3.76133500 -0.23784500

B -5.84821700 3.71932700 -0.54287200

N 5.05717100 4.87701100 0.57937200

N 0.67456600 4.90142700 -0.01842100

N -3.69003000 4.86420100 -0.55670100

N -8.16962500 4.79746500 -0.78777200

H 7.89229800 -5.90727000 -0.92927600

H 9.33624000 -4.83696600 -0.68532000

H 9.12491500 -2.18599700 -0.20189400

H 8.93143900 0.12109000 0.18381000

H 9.02267500 2.23169800 0.53875500

H 7.73768800 6.02529700 1.00694000

H 9.21448700 4.98607000 0.96180500

H 5.62152400 5.70626500 0.73225400

H 3.38506300 6.04391700 0.55988300

H 1.23114400 5.74663000 0.03910900

H -0.99401700 6.05268600 -0.20637700

H -3.13324200 5.70828300 -0.61749400

H -5.37913500 5.98453400 -0.78904000

H -9.14549300 4.65140100 -1.00723500

H -7.76229400 5.59414900 -1.25182400

H -9.01577200 2.32651600 -0.34647800

H -9.17494700 -0.01541200 0.13182600

H -8.87411600 -2.34148300 0.56351600

H -9.23835600 -4.61653300 1.02045800

H -7.77370300 -5.74473400 1.08140400

H -5.39491900 -6.02501200 0.82561300

H -3.00151000 -5.83174000 0.46256600

H -1.03002800 -6.06355900 -0.19427600

H 1.33542100 -5.81475500 -0.59118700

H 5.76544900 -5.72012800 -0.96089600

H 3.36759500 -5.97325500 -0.99433100

Na 0.09227000 -1.51486500 2.06847900

1H_2_$Na/BC_4_N

C -8.04354700 -2.30114900 -0.24981700

C -8.22632100 -4.89736700 -0.68405200

C -7.93411500 2.46282400 0.46680400

C -7.83926300 0.10460700 0.11721300

C -5.80817600 -1.22702600 -0.17215200

C -7.23655400 -1.16096800 -0.10360600

C -5.84597200 -3.61599800 -0.55426800

C -3.77696600 -4.95935700 -0.79460900

C -5.13636700 -4.82852900 -0.77367500

C -8.10503400 5.05361900 0.83384800

C -5.74703000 3.76776800 0.46627000

C -5.05805000 2.52562000 0.26939400

C -5.80248200 1.32778300 0.17455900

C -3.55856500 0.07470100 -0.09592900

C -2.92197400 -1.21008900 -0.24485600

C -3.71024400 -2.40547300 -0.39353800

C -1.43906600 -3.68440000 -0.46910300

C -0.72813000 -4.91617300 -0.45389000

C -3.68921100 5.04367900 0.41810400

C -3.62405400 2.49819800 0.16031100

C -1.48335000 1.31421500 -0.05425000

C -1.48614200 -1.27426200 -0.23845300

C 0.66510100 -2.46560900 -0.17269100

C 0.61273200 -5.04409200 -0.23874400

C -1.35913700 3.76919900 0.02547100

C 0.73309100 2.46903200 -0.14757300

C -0.70175000 2.50657700 -0.05505600

C 0.79039100 0.03449000 -0.14588300

C 2.87891200 -1.31582000 -0.00563500

C 1.44939800 -1.24658900 -0.11281200

C 2.92559900 -3.73569100 0.14888800

C 4.97726200 -5.04629800 0.55370400

C 3.61884900 -4.94227300 0.36520300

C 0.68756600 5.03435100 -0.18605500

C 3.00682900 3.72069000 -0.37513400

C 3.66319500 2.46893900 -0.27693900

C 2.87037000 1.27628700 -0.16717600

C 5.12801900 -0.00117500 -0.02070100

C 5.75836400 -1.24490000 0.15791900

C 5.00996400 -2.48404000 0.25135700

C 7.32135800 -3.71923900 0.60971400

C 8.16817400 -4.75924300 0.83807000

C 5.05145800 4.97560300 -0.62513400

C 5.10471200 2.42683200 -0.29643300

C 7.22149100 1.25731900 -0.12952100

C 7.19519600 -1.29759800 0.24591700

C 7.39947200 3.70124400 -0.49429900

C 7.95433900 2.44573400 -0.29836200

B -7.39912700 -3.63404600 -0.50215000

B -2.96292400 -3.70137400 -0.57485700

B 1.41343400 -3.76761700 -0.07691600

B 5.76632700 -3.78016100 0.48118600

N -5.12935300 -2.42201300 -0.37710900

N -0.75954000 -2.47961000 -0.31243700

N 3.60674200 -2.51692900 0.12807600

N 7.86836700 -2.45303600 0.45784800

B -5.05867100 0.05271800 -0.03077700

B -0.72835200 0.02730000 -0.15946800

B 3.61975700 -0.01294700 -0.06361500

B 7.98953100 -0.02703300 0.08827100

N -7.20426600 1.30608500 0.25620300

N -2.89162700 1.29374500 0.00074800

N 1.46983300 1.26060800 -0.15479000

N 5.82007700 1.22424500 -0.14791700

B -7.29523200 3.80589900 0.60025800

B -2.87474700 3.80933600 0.20538100

B 1.49141200 3.77406000 -0.24549200

B 5.86986600 3.73124100 -0.48072800

N -5.03166800 4.91249300 0.53264600

N -0.64859400 4.91940000 -0.04649200

N 3.71496800 4.87174200 -0.55097600

N 8.19953600 4.78887200 -0.68713400

H -7.85100800 -5.90235800 -0.87173100

H -9.31491700 -4.85981700 -0.63608900

H -9.12221100 -2.15467100 -0.17936200

H -8.91898600 0.16202200 0.18052000

H -9.00344600 2.27496900 0.51360100

H -7.71362000 6.06763200 0.94705100

H -9.19092200 5.02865100 0.91334700

H -5.59407300 5.74397400 0.68039300

H -3.35496300 6.07518500 0.51099600

H -1.20439800 5.76543000 0.00582600

H 1.02623100 6.06733000 -0.22061200

H 3.15463500 5.71111900 -0.64046700

H 5.40196300 5.99233600 -0.78512300

H 9.19826600 4.72137200 -0.56151500

H 7.81126300 5.71655100 -0.68287700

H 9.03558900 2.32247900 -0.26864400

H 9.18632400 -0.02865600 0.13316900

H 8.87812400 -2.35868200 0.50369300

H 9.24645200 -4.62876000 0.90700800

H 7.78768900 -5.76524200 0.95792300

H 5.39131400 -6.03938100 0.72061200

H 2.99628400 -5.83154000 0.38015400

H 1.02159700 -6.05217000 -0.23332000

H -1.34686700 -5.79509200 -0.60178700

H -5.77256000 -5.69495100 -0.92398100

H -3.37633800 -5.95528700 -0.97376200

Na -0.20703800 -1.57464300 2.05198600

H -2.05673800 -3.28922300 2.30406000

H -1.87703700 -3.15396500 3.01852200

2H_2_$Na/BC_4_N

C -8.03879700 -2.30188100 -0.30159400

C -8.22008200 -4.89446300 -0.75616300

C -7.93193600 2.45652400 0.45339600

C -7.83598400 0.10110400 0.08476200

C -5.80381700 -1.22886200 -0.20498600

C -7.23237800 -1.16291600 -0.14285200

C -5.83972400 -3.61553500 -0.60133900

C -3.76954600 -4.95895600 -0.83202200

C -5.12899200 -4.82748800 -0.82094300

C -8.10371400 5.04414000 0.84181300

C -5.74470400 3.76141000 0.47010300

C -5.05527500 2.52077200 0.26517800

C -5.79938700 1.32365700 0.15891400

C -3.55471600 0.07245200 -0.11198200

C -2.91726300 -1.21184200 -0.26500700

C -3.70485700 -2.40641800 -0.42207300

C -1.43342500 -3.68570500 -0.48535500

C -0.72252200 -4.91745600 -0.46359000

C -3.68662000 5.03764700 0.43621800

C -3.62100300 2.49427500 0.15913900

C -1.47958300 1.31186900 -0.06182200

C -1.48168900 -1.27571000 -0.25459300

C 0.66865500 -2.46650300 -0.17913400

C 0.61703000 -5.04504100 -0.23932200

C -1.35592700 3.76684900 0.03335800

C 0.73639400 2.46816500 -0.15129200

C -0.69836900 2.50490900 -0.05585000

C 0.79329400 0.03331000 -0.15770900

C 2.88138900 -1.31599500 -0.01003400

C 1.45205700 -1.24729800 -0.11996000

C 2.92792700 -3.73486300 0.15731800

C 4.97824900 -5.04192000 0.58069800

C 3.62066600 -4.93975100 0.38490600

C 0.69019700 5.03344100 -0.17800600

C 3.00950300 3.72025400 -0.38338800

C 3.66529500 2.46723800 -0.29689500

C 2.87280000 1.27497600 -0.18355000

C 5.13023300 -0.00201600 -0.03514200

C 5.76043900 -1.24404500 0.15530200

C 5.01191100 -2.48210400 0.25844500

C 7.32204800 -3.71316800 0.63528000

C 8.16818400 -4.75078300 0.87763800

C 5.05388500 4.97336100 -0.64658900

C 5.10652900 2.42341700 -0.32822500

C 7.22366800 1.25470000 -0.16000800

C 7.19716700 -1.29548400 0.24587300

C 7.39976600 3.69032200 -0.57691200

C 7.95571000 2.44011100 -0.35696100

B -7.39324700 -3.63310800 -0.55972600

B -2.95653500 -3.70201300 -0.60231000

B 1.41677500 -3.76817200 -0.07624200

B 5.76757900 -3.77617300 0.50165200

N -5.12403200 -2.42277900 -0.41353300

N -0.75494300 -2.48081500 -0.32509200

N 3.60894200 -2.51613300 0.13169100

N 7.86964000 -2.44875500 0.47185200

B -5.05504100 0.04997000 -0.05244400

B -0.72485200 0.02572500 -0.17459000

B 3.62224600 -0.01386800 -0.07692800

B 7.99168300 -0.02680700 0.07258900

N -7.20157400 1.30134400 0.23569700

N -2.88852000 1.29088800 -0.00711000

N 1.47263000 1.25938200 -0.16544700

N 5.82224400 1.22183200 -0.17379500

B -7.29336000 3.79835700 0.60008800

B -2.87164800 3.80528700 0.21471300

B 1.49466300 3.77370200 -0.24529800

B 5.87140900 3.72508100 -0.53066700

N -5.02943500 4.90540200 0.54732800

N -0.64546700 4.91744100 -0.03446600

N 3.71847700 4.87180100 -0.55365100

N 8.19980600 4.78346000 -0.76085300

H -7.84421300 -5.89815600 -0.94957700

H -9.30892700 -4.85655200 -0.71465300

H -9.11780400 -2.15574900 -0.23582000

H -8.91596200 0.15808700 0.14409500

H -9.00143500 2.26845100 0.49527100

H -7.71246300 6.05708800 0.96475200

H -9.18982900 5.01867400 0.91794400

H -5.59212200 5.73568600 0.70063600

H -3.35256700 6.06835100 0.53816800

H -1.20133000 5.76315000 0.02261100

H 1.02807100 6.06673600 -0.21068800

H 3.15985700 5.71464300 -0.61765200

H 5.40651500 5.99286300 -0.78132000

H 9.18841900 4.65979100 -0.92390000

H 7.80409900 5.63484900 -1.12365700

H 9.03714200 2.31677100 -0.33340300

H 9.18855800 -0.02848000 0.11506600

H 8.87929500 -2.35404800 0.51932300

H 9.24617900 -4.61947700 0.94929500

H 7.78725600 -5.75546400 1.00680500

H 5.39172800 -6.03366000 0.75676000

H 2.99819800 -5.82907900 0.40296700

H 1.02596900 -6.05308100 -0.22927800

H -1.34033600 -5.79665100 -0.61400800

H -5.76446200 -5.69298000 -0.97952300

H -3.36790300 -5.95432200 -1.01211000

Na -0.20915900 -1.58002400 2.05293300

H -0.65157900 0.34013800 3.74375900

H -0.78869900 0.65277200 3.07920000

H -2.02107200 -3.34847200 2.27872000

H -1.84865100 -3.21517400 2.99515900

3H_2_$Na/BC_4_N

C -8.04753500 -2.30657500 -0.28681300

C -8.25328800 -4.88041200 -0.72051000

C -7.95295800 2.45104500 0.43336500

C -7.85158700 0.09408400 0.08259400

C -5.81320700 -1.23719500 -0.19648400

C -7.23834800 -1.16568400 -0.13451800

C -5.84564400 -3.62716100 -0.57872700

C -3.77679600 -4.96630400 -0.80758700

C -5.13848400 -4.83782100 -0.79168300

C -8.12640400 5.04303500 0.80071100

C -5.76705300 3.75399200 0.44269500

C -5.07358000 2.51515500 0.24839500

C -5.81660800 1.31481000 0.15018100

C -3.56522700 0.06548200 -0.11165300

C -2.92738600 -1.21555500 -0.25963200

C -3.71511500 -2.41466000 -0.40948000

C -1.43880500 -3.68823400 -0.48895800

C -0.72465500 -4.91597900 -0.48876900

C -3.71342900 5.03431700 0.40102000

C -3.63938000 2.48984100 0.14316800

C -1.49583200 1.31037100 -0.07231800

C -1.49159600 -1.27791800 -0.25657100

C 0.66199500 -2.46344300 -0.21006700

C 0.62028500 -5.04015700 -0.29194200

C -1.37800000 3.76570000 0.01429400

C 0.71784800 2.47223500 -0.16631600

C -0.71624500 2.50673400 -0.07081200

C 0.78144400 0.03506800 -0.17648700

C 2.87523600 -1.30854000 -0.04783200

C 1.44358000 -1.24260100 -0.15350400

C 2.93028400 -3.72937600 0.09567200

C 4.98516200 -5.03416700 0.49975800

C 3.62671300 -4.93381800 0.30592500

C 0.66317500 5.03664200 -0.19279400

C 2.98855000 3.73202800 -0.38725100

C 3.64816500 2.47891300 -0.30473400

C 2.85663200 1.28514500 -0.20088300

C 5.11907500 0.01210600 -0.05478800

C 5.75733100 -1.22910900 0.12682500

C 5.01105600 -2.46900500 0.21255200

C 7.32540000 -3.70181500 0.58520400

C 8.16377400 -4.74661600 0.81959300

C 5.03347800 4.98734300 -0.62631200

C 5.08962300 2.43510300 -0.32961100

C 7.21251500 1.27009300 -0.15359100

C 7.19402100 -1.27574300 0.22576100

C 7.38274300 3.71097500 -0.56015400

C 7.93905100 2.46866600 -0.34236700

B -7.39811300 -3.63594100 -0.53520400

B -2.96504400 -3.70909200 -0.58819200

B 1.41814400 -3.76397000 -0.12681000

B 5.77227800 -3.76655300 0.44150300

N -5.13218400 -2.43387700 -0.39861500

N -0.76157400 -2.48261900 -0.33457700

N 3.60850100 -2.50793700 0.08289900

N 7.86901500 -2.43433700 0.44291300

B -5.06669800 0.04223100 -0.05148600

B -0.73672100 0.02389100 -0.18413500

B 3.61379200 -0.00491500 -0.10257200

B 7.98015900 -0.00415700 0.07228900

N -7.21836900 1.29557800 0.22568700

N -2.90147100 1.28600400 -0.01512600

N 1.45892000 1.26420300 -0.18237900

N 5.80761200 1.23530100 -0.17873000

B -7.31489300 3.79231000 0.57015200

B -2.89281600 3.80118700 0.19011000

B 1.47470200 3.77845500 -0.25777400

B 5.85463700 3.74076700 -0.51990800

N -5.05422900 4.90167800 0.51167900

N -0.67013900 4.91925400 -0.05297800

N 3.69645000 4.88461200 -0.54228400

N 8.17723300 4.82478300 -0.72498800

H -7.89789100 -5.89249100 -0.90815800

H -9.34053300 -4.81791600 -0.67573200

H -9.12665500 -2.16209900 -0.22311500

H -8.93172600 0.14731300 0.13957300

H -9.02190300 2.26051900 0.47447100

H -7.73514200 6.05657800 0.91637000

H -9.21255600 5.01812500 0.87492500

H -5.61831300 5.73238900 0.65744800

H -3.38065900 6.06622200 0.49427500

H -1.22694600 5.76448700 0.00151800

H 1.00002700 6.07022700 -0.22630200

H 3.13980600 5.72904700 -0.59895700

H 5.38705600 6.00852300 -0.74381800

H 9.15516200 4.68235200 -0.93767600

H 7.77378300 5.62724100 -1.18249500

H 9.02092000 2.34747200 -0.31358600

H 9.17794800 -0.00138100 0.12279000

H 8.87718700 -2.33562800 0.49851500

H 9.24176000 -4.62134600 0.90151000

H 7.77747800 -5.75129200 0.93146600

H 5.40183300 -6.02690300 0.66186400

H 3.00761300 -5.82578400 0.31325600

H 1.03299000 -6.04659200 -0.29920700

H -1.34099800 -5.79705800 -0.63451800

H -5.77165300 -5.70724600 -0.94239600

H -3.37460300 -5.96216400 -0.98262400

Na -0.07025200 -1.52796300 2.05988900

H 2.29880400 -0.96787600 2.82427400

H 2.00755500 -1.06261000 3.50613600

H -1.01910000 0.29083300 3.71251000

H -1.12345600 0.59297200 3.03766600

H -1.76844300 -3.42941700 2.28107800

H -1.61190700 -3.28445200 2.99840900

4H_2_$Na/BC_4_N

C -8.04417200 -2.32821800 -0.30870600

C -8.22336000 -4.91351400 -0.78137800

C -7.97522300 2.42438800 0.48520300

C -7.85827900 0.07251400 0.09613700

C -5.81759700 -1.23916100 -0.21811000

C -7.24359300 -1.18450800 -0.14576100

C -5.83889600 -3.62168200 -0.63660900

C -3.76052100 -4.94624200 -0.90167100

C -5.12142600 -4.82511800 -0.87489300

C -8.16135500 5.00997600 0.89509600

C -5.79683000 3.74360900 0.49862900

C -5.09833600 2.51044200 0.27958300

C -5.83283100 1.30847900 0.16786700

C -3.57872500 0.07887800 -0.12876900

C -2.93295100 -1.19795800 -0.29973900

C -3.71229500 -2.39782600 -0.46294000

C -1.43080000 -3.65766000 -0.56313300

C -0.70983600 -4.88337200 -0.56409500

C -3.74892900 5.03740300 0.46369000

C -3.66383200 2.49676900 0.16507500

C -1.51384500 1.33400600 -0.07768500

C -1.49634000 -1.25103000 -0.30009700

C 0.66284400 -2.42536300 -0.25320800

C 0.63211000 -5.00357100 -0.34808000

C -1.40958200 3.78867000 0.03909300

C 0.69230500 2.50808900 -0.16467100

C -0.74244100 2.53263900 -0.06381200

C 0.76809600 0.07420100 -0.19715300

C 2.86651700 -1.26012900 -0.06990700

C 1.43578000 -1.20193000 -0.17732600

C 2.93012000 -3.68005800 0.07439500

C 4.99119800 -4.97620100 0.48485800

C 3.63125900 -4.88142200 0.29575900

C 0.62593300 5.07299200 -0.16789400

C 2.95468300 3.77954000 -0.39227500

C 3.62090200 2.53148100 -0.31303400

C 2.83794000 1.33219100 -0.21187300

C 5.10544100 0.07050400 -0.08528100

C 5.74530200 -1.16931600 0.08436000

C 5.00625000 -2.41337500 0.17735500

C 7.32765400 -3.63317000 0.52070100

C 8.18331900 -4.66859900 0.73859800

C 4.98842600 5.05150700 -0.64617000

C 5.06283700 2.50007600 -0.34268700

C 7.18933800 1.34455700 -0.20089600

C 7.18356900 -1.21210900 0.16318000

C 7.34661400 3.79249000 -0.54824600

C 7.91220500 2.53962200 -0.36688200

B -7.39130300 -3.64919200 -0.58212900

B -2.95537200 -3.68584400 -0.66619900

B 1.42060800 -3.72252000 -0.16870700

B 5.77241700 -3.70499600 0.40413700

N -5.13138700 -2.42517100 -0.44293100

N -0.76109500 -2.44958700 -0.39089600

N 3.60345100 -2.45558300 0.05724500

N 7.86552700 -2.36357100 0.36734700

B -5.07900000 0.04367000 -0.05933500

B -0.75010900 0.05492200 -0.20853500

B 3.59732600 0.04815100 -0.12221000

B 7.96810200 0.06455700 0.00414600

N -7.23464500 1.27340700 0.25288500

N -2.92282800 1.30211300 -0.01593600

N 1.43789300 1.30503000 -0.19246700

N 5.78816400 1.30152100 -0.20904600

B -7.34548800 3.76676700 0.63802800

B -2.92522700 3.81413200 0.22788300

B 1.43974700 3.82008500 -0.25011700

B 5.81700200 3.81161500 -0.52121900

N -5.09104100 4.89241900 0.58097200

N -0.70815500 4.94497900 -0.02101200

N 3.65350100 4.93725200 -0.56141000

N 8.13713700 4.88741200 -0.73925300

H -7.84864300 -5.91574400 -0.98437800

H -9.31165300 -4.87457300 -0.73055800

H -9.12368100 -2.19034500 -0.23392200

H -8.93848200 0.11822200 0.16259100

H -9.04251500 2.22574900 0.53193800

H -7.77351300 6.02353400 1.02366800

H -9.24686300 4.97924600 0.97766700

H -5.65972500 5.71668600 0.74383600

H -3.42356300 6.07023100 0.57196800

H -1.27056700 5.78564400 0.04655100

H 0.95594200 6.10910000 -0.19004300

H 3.08650100 5.77355100 -0.63722500

H 5.33025500 6.07232600 -0.79858100

H 9.13729200 4.82628900 -0.62196400

H 7.74219100 5.81216600 -0.72414500

H 8.99452700 2.42411300 -0.34668800

H 9.16515500 0.07058300 0.03995800

H 8.87502500 -2.26288500 0.40603900

H 9.26124100 -4.53127900 0.79882900

H 7.81040200 -5.67734500 0.85909700

H 5.41172700 -5.96653700 0.65163400

H 3.01445600 -5.77476000 0.31075900

H 1.04959700 -6.00816200 -0.35527200

H -1.32118600 -5.76521400 -0.72511300

H -5.75139500 -5.69459700 -1.03594200

H -3.35335700 -5.93661200 -1.09616800

Na -0.23178200 -1.59159500 2.01018200

H 1.68676000 -2.98436400 2.89830000

H 1.51965000 -2.66575900 3.55358900

H -0.78991900 0.35114000 3.65357300

H -0.91370500 0.64749000 2.97887200

H -2.23531800 -3.17983500 2.23206600

H -2.04307200 -3.07231000 2.94743000

H 4.68482700 -5.01441100 3.53407400

H 4.64017100 -5.12302100 4.26876600
